# Transcriptional Repression of Ferritin Light Chain Increases Ferroptosis Sensitivity in Lung Adenocarcinoma

**DOI:** 10.3389/fcell.2021.719187

**Published:** 2021-10-26

**Authors:** Yikun Wang, Shiyu Qiu, Hong Wang, Jiangtao Cui, Xiaoting Tian, Yayou Miao, Congcong Zhang, Leiqun Cao, Lifang Ma, Xin Xu, Yongxia Qiao, Xiao Zhang

**Affiliations:** ^1^Shanghai Institute of Thoracic Oncology, Shanghai Chest Hospital, Shanghai Jiao Tong University, Shanghai, China; ^2^School of Public Health, Shanghai Jiao Tong University School of Medicine, Shanghai, China; ^3^Department of Thoracic Surgery, Shanghai Chest Hospital, Shanghai Jiao Tong University, Shanghai, China; ^4^School of Medicine, Anhui University of Science and Technology, Huainan, China

**Keywords:** YAP, TFCP2, erastin, labile iron, LUAD, lipid peroxidation

## Abstract

Ferroptosis is an iron- and lipid peroxidation-dependent form of regulated cell death. The release of labile iron is one of the important factors affecting sensitivity to ferroptosis. Yes-associated protein (YAP) controls intracellular iron levels by affecting the transcription of *ferritin heavy chain (FTH)* and *transferrin receptor (TFRC)*. However, whether YAP regulates iron metabolism through other target genes remains unknown. Here, we observed that the system Xc^–^ inhibitor erastin inhibited the binding of the WW domain and PSY motif between YAP and transcription factor CP2 (TFCP2), and then suppressed the transcription of *ferritin light chain (FTL)* simultaneously mediated by YAP, TFCP2 and forkhead box A1 (FOXA1). Furthermore, inhibition of FTL expression abrogated ferroptosis-resistance in cells with sustained YAP expression. Unlike *FTH*, which exhibited first an increase and then a decrease in transcription, *FTL* transcription continued to decline after the addition of erastin, and a decrease in lysine acetyltransferase 5 (KAT5)-dependent acetylation of *FTL* was also observed. In lung adenocarcinoma (LUAD) tissues, lipid peroxidation and labile iron decreased, while YAP, TFCP2 and FTL increased compared to their adjacent normal tissues, and the lipid peroxidation marker 4-hydroxynonenal (4-HNE) was negatively correlated with the level of FTL or the degree of LUAD malignancy, but LUAD tissues with lower levels of 4-HNE showed a higher sensitivity to ferroptosis. In conclusion, the findings from this study indicated that the suppression of *FTL* transcription through the inhibition of the YAP-TFCP2-KAT5 complex could be another mechanism for elevating ferroptosis sensitivity and inducing cell death, and ferroptotic therapy is more likely to achieve better results in LUAD patients with a lower degree of lipid peroxidation.

## Introduction

In 2012, Stockwell et al. described ferroptosis, a type of metabolism-related cell death that is elicited by inhibiting system Xc^–^ using erastin, a potent ferroptotic agonist (Dixon et al., 2012). System Xc^–^ inhibition suppresses cystine uptake and downstream glutathione (GSH) synthesis, while simultaneously leading to the elevation of labile iron levels (Wang et al., 2020). The release of labile iron is closely related to ferroptosis sensitivity (Fuhrmann et al., 2020; Zhang et al., 2021). A common mechanism for ferroptosis agonist-induced iron release is nuclear receptor coactivator 4 (NCOA4)-mediated autophagic degradation of ferritin, referred to as ferritinophagy (Mancias et al., 2014). Our research group also reported a mechanism that affected ferroptosis sensitivity caused by an increase in labile iron due to a disruption of the transcription balance of *FTH* (Zhang et al., 2021). However, whether other mechanisms by which labile iron influences ferroptosis sensitivity remains to be explored.

Lung cancer is the leading cause of cancer-related death worldwide, with LUAD being the most prevalent subtype (Herbst et al., 2018; Sayin et al., 2019). Although therapeutic approaches have been developed for some subtypes of LUAD, the overall survival of LUAD patients remains low (Herbst et al., 2018; Jin et al., 2019); hence, effective treatment strategies for LUAD need to be developed. YAP, a downstream transcription activator of Hippo signaling, functions as a proto-oncoprotein in several malignancies, including LUAD (Zhao et al., 2007, 2008; Zanconato et al., 2016). In serine/threonine kinase 11 (STK11)-deficient LUAD, YAP directly stimulates downstream effector surviving to promote malignant progression, and YAP overexpression in the *Kras*^G12D^ lung cancer mouse model accelerates tumor progression (Zhang et al., 2015). Targeting YAP or YAP-related factors exerts a tumor-suppressive effect in LUAD. For example, angiomotin (AMOT) absorbs YAP to inhibit growth factor cysteine-rich angiogenic inducer 61 (Cyr61) and reduce tumor growth *in vivo* (Hsu et al., 2015). Vestigial-like family member 4 (VGLL4) competes with YAP in binding to TEA domain family members (TEADs) and thus inhibits tumor growth in LUAD (Zhang et al., 2014). Strategies targeting YAP might present novel treatment options for LUAD.

In addition to serving as a transcription coactivator, YAP is highly responsive to environmental conditions and is a master metabolic regulator (Koo and Guan, 2018). During glucose metabolism, YAP stimulates glucose transporter type 3 (GLUT3) transcription to enhance glucose uptake (Wang W. et al., 2015) and promotes glycolysis by stimulating the glycolytic enzymes hexokinase 2 (HK2) and 6-phosphofructo-2-kinase/fructose-2,6-biphosphatase 3 (PFKFB3) (Zheng et al., 2017), as well as the hexosamine biosynthesis pathway (HBP) *via* the activation of nudix hydrolase 9 (NUDT9) and solute carrier family 5 member 3 (SLC5A3) (Zhang et al., 2017a). HBP activation leads to increased YAP O-linked beta-N-acetylglucosaminylation (O-GlcNAcylation), contributing to a vicious cycle of cancer progression (Zhang et al., 2017a). YAP is also an activator of the glutamate-related aminotransferases glutamic-oxaloacetic transaminase 1 (GOT1) and phosphoserine aminotransferase 1 (PSAT1), which mediate the conversion of glutamate to α-ketoglutaric acid (α-KG), thereby stimulating cancer cell growth (Yang et al., 2018). YAP is also a critical regulator for ferroptosis. On the one hand, YAP is a transcriptional stimulator of the ferroptosis-activation genes *acyl-CoA synthetase long chain family member 4* (*ACSL4)* and *TFRC* (Wu et al., 2019); on the other hand, the decrease in YAP caused by ferroptosis agonists inhibited *FTH* transcription mediated by TFCP2, and ferroptosis is further boosted by labile iron production (Zhang et al., 2021). However, it remains to be elucidated whether the YAP-TFCP2 transcription complex plays a regulatory role in *FTL* transcription and whether ferroptosis sensitivity is modulated by the transcription of *FTL*.

Here, we discovered that system Xc^–^ inhibition, such as erastin treatment, suppressed the interaction between the YAP WW domain and the TFCP2 PSY motif and further inhibited the *FTL* transcription mediated by YAP, TFCP2 and forkhead box A1 (FOXA1). In addition, FTL knockdown abrogated sustained YAP induced ferroptosis inhibition, implying that FTL could be another ferroptosis sensitivity regulator. Finally, we observed that the tumor tissues from LUAD patients with lower 4-HNE levels exhibited higher ferroptosis sensitivity, and FTL significantly decreased after piperazine erastin (PKE) treatment. In summary, this study suggests new ferroptosis sensitivity markers and strategies for increasing the ferroptotic treatment effect on LUAD.

## Results

### Interaction Between Yes-Associated Protein and Transcription Factor CP2 Is Inhibited by Erastin

**FIGURE 1:**
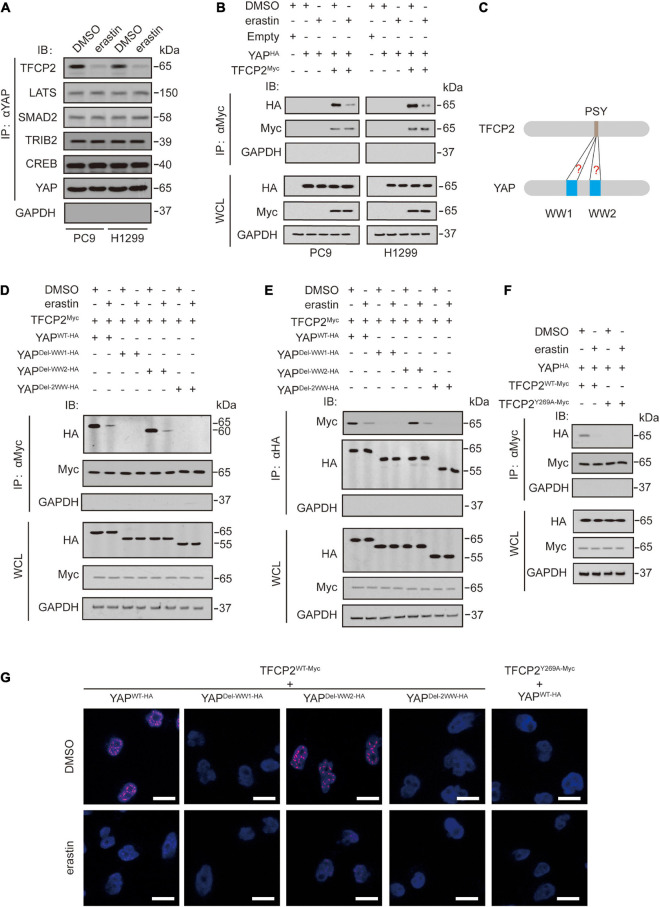
Erastin inhibits YAP and TFCP2 interaction. **(A)** A Co-IP experiment was performed using anti-YAP antibodies in control cells and PC9 or H1299 cells with or without erastin (10 μM, 24 h) treatment. The YAP level in each co-IP samples was adjusted to the same protein content. The interaction between YAP and TFCP2, LATS, SMAD2, TRIB2, and CREB were measured using the indicated antibodies by IB. **(B)** A Co-IP experiment was performed using anti-Myc antibodies in control cells and PC9 or H1299 cells with the indicated vectors transfected before erastin (5 μM, 4 h) treatment. The indicated proteins in co-IP samples or WCL were measured by IB. **(C)** Schematic representation for interactions between the PSY motif of TFCP2 and the WW domains of YAP. **(D–F)** Co-IP experiments were performed using anti-Myc **(D,F)** and anti-HA **(E)** antibodies in PC9 cells with the indicated vectors transfected before erastin (5 μM, 4 h) treatment. The TFCP2-Myc **(D,F)** and YAP-HA **(E)** level in each co-IP samples was adjusted to the same protein content. The indicated proteins in co-IP samples or WCL were measured by IB. **(G)** PLA experiments were performed using anti-Myc and anti-HA antibodies in PC9 cells with indicated vectors transfected before erastin treatment (5 μM, 4 h). Scale bar, 50 μm. Images of IB are representative ones of 3 independent experiments.

Erastin inhibits YAP expression by suppressing its O-GlcNAcylation and recruiting its specific ubiquitin E3 ligase beta-transducin repeat-containing homolog protein (βTRCP) in LUAD cells (Zhang et al., 2021). We investigated whether erastin inhibits other YAP upstream regulators, such as TFCP2 (Zhang et al., 2017b), large tumor suppressor kinase (LATS) (Hao et al., 2008), SMAD family member 2 (SMAD2) (Grannas et al., 2015), tribbles pseudokinase 2 (TRIB2) (Wang et al., 2013b), and cAMP responsive element binding protein (CREB) (Wang et al., 2013a). We observed that erastin specifically inhibited the binding of endogenous TFCP2 to YAP in PC9 and H1299 cells (Figure 1A and Supplementary Figure 1A). Additionally, the binding between exogenous TFCP2-Myc and YAP-HA was inhibited by erastin treatment (Figure 1B and Supplementary Figure 1B). These findings suggested that erastin abolishes the interaction between YAP and TFCP2 in PC9 and H1299 cells. Previous studies have reported that the interaction between TFCP2 and YAP depends on the PSY-WW interaction (Zhang et al., 2017b; Figure 1C). Through coimmunoprecipitation (co-IP) and protein ligation assay (PLA) experiments, we observed that the first WW domain of YAP was essential for the binding between TFCP2 and YAP, and this binding was suppressed by erastin treatment (Figures 1D,E,G and Supplementary Figures 1C,D). Moreover, erastin blocked the interaction between YAP and TFCP2, and the TFCP2 Y269A (Y for tyrosine, A for alanine) mutation altered its PSY motif, abrogating the interaction between YAP and TFCP2; this finding suggested that the PSY motif is essential for the interaction between YAP and TFCP2, and this interaction could be inhibited by erastin treatment (Figures 1F–G and Supplementary Figures 1E–G). These findings suggested that erastin blocks the YAP-TFCP2 interaction by abolishing the PSY-WW interaction.

Furthermore, we investigated the mechanisms by which erastin inhibits the interaction between YAP and TFCP2. In our previous study, we reported that when system Xc^–^ is suppressed, endogenous glutamate accumulates, which promotes Ca^2+^-dependent cyclic adenosine monophosphate (cAMP) production by adenylate cyclase 10 (ADCY10) to stimulate protein kinase A (PKA)-associated phosphorylation and suppression of glutamine-fructose-6-phosphate transaminase (GFPT1). Subsequently, YAP is inevitably suppressed (Zhang et al., 2021). We speculated that the inhibition of the YAP-TFCP2 interaction might also be controlled by a similar mechanism. We used a doxycycline (Dox)-inducible GGG cell model (in which GLUD1 overexpression (G) and GLAST (G) and GLT1 knockdown (G) were simultaneously achieved) to block glutamate uptake and stimulate glutamate degradation (Zhang et al., 2021), and we found that GGG cells could reverse erastin-induced inhibition of the YAP-TFCP2 interaction, but this effect could be further reversed by the addition of epigallocatechol gallate (EGCG), a GLUD1 inhibitor (Pournourmohammadi et al., 2017; Supplementary Figure 1H). Additionally, erastin-induced inhibition of the YAP-TFCP2 interaction could be reversed by the calcium chelator ethylenebis (oxyethylenenitrilo) tetraacetic acid (EGTA) (Supplementary Figure 1I), the knockout of ADCY10 (Supplementary Figure 1J), H89 and Rp-cAMPs (two PKA inhibitors (Botelho et al., 1988; Chijiwa et al., 1990)) (Supplementary Figure 1K), XBP1s (a transcription factor that controls the transcription of GFPT1 (Chen et al., 2014)) (Supplementary Figure 1L), and N-acetylglucosamine (GlcNAc) and glucosamine (GlcN) (two metabolites that can bypass glucosamine-6-phosphate(GlcN-6-P) and N-acetylglucosamine-6-phosphate (GlcNAc-6-P) to replace GFPT1 function in HBP (Moloughney et al., 2016)), whereas glucose did not have this effect (Supplementary Figure 1M). These findings suggested that erastin-induced inhibition of the YAP-TFCP2 interaction was controlled by the previously reported ADCY10/PKA/GFPT1 axis (Zhang et al., 2021).

### Yes-Associated Protein/Transcription Factor CP2 Suppression Elevates Labile Iron via Ferritin Light Chain Inhibition

**FIGURE 2:**
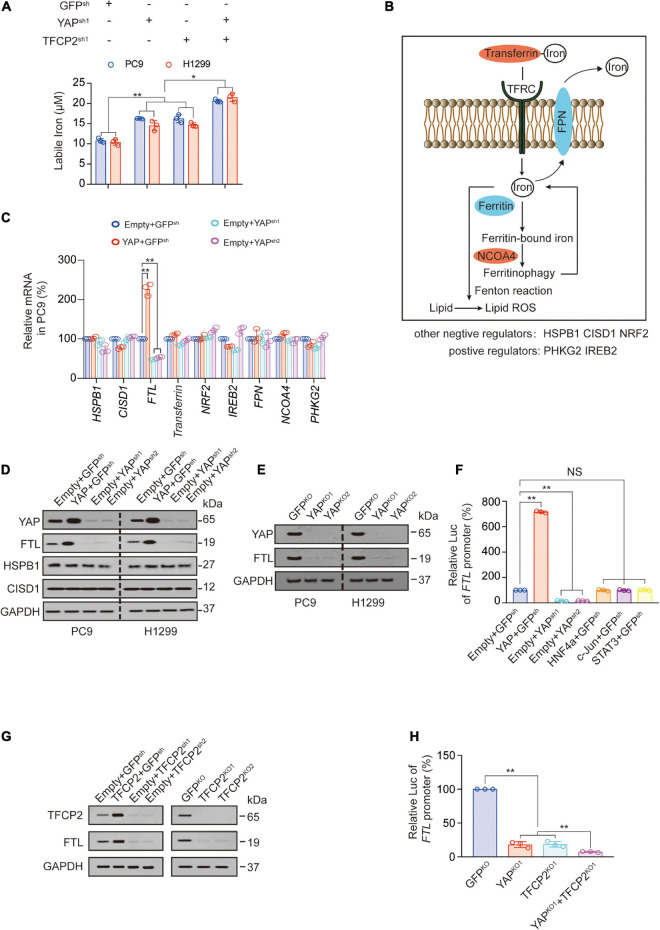
YAP and TFCP2 suppression upregulates labile iron through inhibiting FTL. **(A)** Labile iron was measured in control cells and PC9 or H1299 cells with YAP and TFCP2 individually or simultaneously knocked out. **(B)** Schematic representation of pathways and regulators that affect ferroptosis *via* labile iron levels. **(C)** The indicated mRNA levels measured in control cells and PC9 cells with YAP overexpression or knockdown. **(D)** YAP, FTL, HSPB1, and CISD1 expressions were measured using the indicated antibodies in control cells and PC9 or H1299 cells with YAP overexpression or knockdown. **(E)** YAP and FTL expressions measured using the indicated antibodies in control cells and PC9 or H1299 cells with YAP knockout. **(F)** *FTL* promoter luciferase (Luc) activity measured in control cells and PC9 cells with the indicated genes overexpression or knockdown. **(G)** TFCP2 and FTL expressions measured using the indicated antibodies in control cells and PC9 cells with TFCP2 overexpression, knockdown or knockout. **(H)** *FTL* promoter Luc activity measured in control cells and PC9 cells with YAP and TFCP2 individually or simultaneously knocked out. The data are shown as the mean ± SD from three biological replicates (including IB). *P < 0.05, **P < 0.01 indicates statistical significance. Data in **(A,C,F,H)** were analyzed using a one-way ANOVA test.

YAP and TFCP2 are two factors affecting ferroptosis (Wu et al., 2019; Zhang et al., 2021). We further characterized the mechanism by which YAP and TFCP2 suppression promotes ferroptosis. At least three metabolites regulate ferroptosis through lipid reactive oxygen species (ROS): phospholipids and labile iron upregulate lipid ROS, whereas GSH downregulates lipid ROS (Dixon and Stockwell, 2014; Yang et al., 2016; Miess et al., 2018; Zhang et al., 2019). We found that YAP and TFCP2 knockdown significantly increased the amount of labile iron in PC9 and H1299 cells, and the simultaneous knockdown of YAP and TFCP2 had a synergistic effect on the rise of labile iron (Figure 2A). However, YAP or TFCP2 knockdown had no regulatory role on the production of GSH and phospholipids (Supplementary Figures 2A,B).

Next, we explored the mechanism by which YAP affects the levels of labile iron. Labile iron is commonly complexed with transferrin and taken up in peripheral tissue by binding to TFRC. FTH and FTL bind to cellular labile iron and are later degraded by ferritinophagy *via* NCOA4. Excess iron can be exported from cells into the circulation by the iron exporter ferroportin (FPN) (Manz et al., 2016; Hassannia et al., 2019; Jung et al., 2019). Other regulators of labile iron in ferroptosis include phosphorylase kinase catalytic subunit gamma 2 (PHKG2) (Yang et al., 2016), iron responsive element binding protein 2 (IREB2) (Dixon et al., 2012), heat shock protein family B member 1 (HSPB1) (Sun et al., 2015), CDGSH iron sulfur domain 1 (CISD1) (Yuan et al., 2016) and nuclear factor erythroid 2 like 2 (NRF2) (Sun et al., 2016; Figure 2B). Since *TFRC* and *FTH* have already been reported as YAP-dependent transcription targets, we further investigated whether other iron-related genes are transcriptionally regulated by YAP. We observed that YAP overexpression significantly promoted the expression of FTL at both the mRNA and protein levels in PC9 and H1299 cells, while YAP knockdown suppressed the expression of FTL (Figures 2C,D and Supplementary Figure 2C). We also observed that knockout of the YAP reduced the expression and promoter activity of *FTL* (Figure 2E and Supplementary Figure 2D). The promoter activities of *FTL* were stimulated by YAP overexpression and repressed by YAP knockdown, without the involvement of other transcription factors such as hepatocyte nuclear factor 4 alpha (HNF4a), c-Jun, and signal transducer and activator of transcription 3 (STAT3) (Figure 2F). Similarly, TFCP2 overexpression promoted FTL expression, while TFCP2 knockdown or knockout suppressed FTL expression (Figure 2G). We also observed the independent and synergistic inhibitory effects of YAP and TFCP2 knockout on *FTL* promoter activity (Figure 2H). These findings suggested that YAP and TFCP2 suppression might upregulate the levels of labile iron by inducing transcriptional repression of *FTL* in LUAD cells.

### Yes-Associated Protein Stimulates *FTL* Transcription via Transcription Factor CP2

**FIGURE 3:**
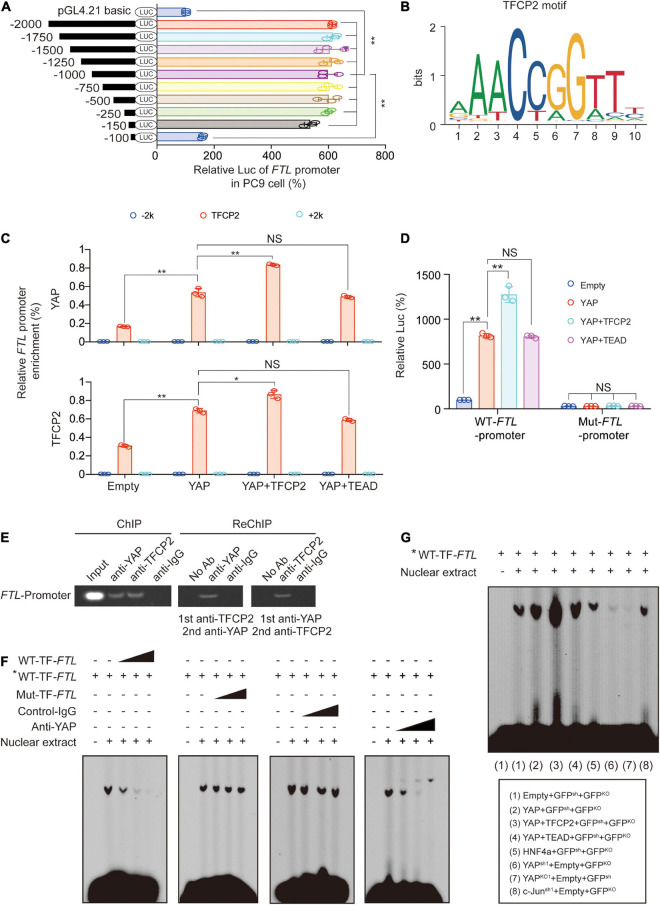
Transcriptions of *FTL* are stimulated by YAP *via* TFCP2. **(A)** The indicated regions of *FTL* promoter Luc activity measured in PC9 cells. **(B)** The TFCP2 motif was searched using the JASPAR database. **(C)** Enrichment of YAP and TFCP2 at the indicated region around the *FTL* promoters in control cells and PC9 cells overexpressing YAP with TFCP2 or TEAD. **(D)** Wild type (WT) or mutant (Mut) *FTL* promoter Luc activity measured in control cells and PC9 cells overexpressing YAP with TFCP2 or TEAD. **(E)** Co-occupancy of YAP and TFCP2 in *FTL* promoters measured by ChIP and Re-ChIP assays using anti-YAP or anti-TFCP2 antibodies in PC9 cells. **(F)** Nuclear extracts from PC9 cells were incubated with WT- or Mut-TF-*FTL* (the TFCP2 motif was mutated in *FTL*) probes with or without the presence of control-IgG or anti-YAP antibodies. The asterisk indicates probes labeled with biotin. The DNA-protein interactions were measured using EMSA. **(G)** Nuclear extracts from PC9 cells with the indicated genes overexpression, knockdown or knockout were incubated with biotin-labeled WT-TF-*FTL*. The DNA-protein interactions were measured using EMSA. The data are shown as the mean ± SD from three biological replicates (including IB). *P < 0.05, **P < 0.01 indicates statistical significance. Data in **(A,C,D)** were analyzed using a one-way ANOVA test.

To investigate the mechanism by which YAP and TFCP2 stimulate the transcription of *FTL*, we constructed a series of luciferase reporters containing truncated versions of the *FTL* promoter to determine whether these regions are essential for the transcription of *FTL*. We found that the activity was abolished after deleting −150∼−100nt of the *FTL* promoter, suggesting that the crucial motif existed in this region (Figure 3A and Supplementary Figure 3A). We used the FIMO tool^1^ to find a significant TFCP2 binding site in the −150∼−100nt region of the *FTL* promoter (Figure 3B and Supplementary Figure 3B). We also found a STAT3 binding site in the *FTL* promoter region and a TFCP2 binding site in the *FTH* promoter region (Supplementary Figure 3B). To verify the possibility that TFCP2 binds to the *FTL* promoter, and to exclude the possibility that STAT3 binds to the *FTL* promoter, we performed chromatin immunoprecipitation (ChIP) experiments. YAP overexpression increased the enrichment of YAP and TFCP2 around the TFCP2 binding motif of the *FTL* promoter, and this effect was further enhanced by simultaneously overexpressing TFCP2 but not TEADs (Figure 3C). The control IgG and STAT3 signals were weak and not dependent on YAP or TFCP2 overexpression, and the “−2k” or “ + 2k” regions around the *FTL* promoter had low levels of YAP and TFCP2 enrichment, demonstrating the specific signal from the TFCP2 binding region (Figure 3C and Supplementary Figures 3C,D). RUNX family transcription factor 2 (Runx2), tumor protein 73 (P73), or SMAD2 did not promote the interaction between YAP and the *FTL* promoter (Supplementary Figure 3D). We mutated the TFCP2 binding motif in the *FTL* promoter region, and observed that these mutations significantly abolished the YAP- and TFCP2-mediated induction of *FTL* promoter activity (Figure 3D). The cooccupancy of YAP and TFCP2 at the *FTL* promoter was also confirmed by ChIP and Re-ChIP experiments using anti-TFCP2 antibodies for the first IP, followed by anti-YAP antibodies for the subsequent IP and *vice versa* (Figure 3E). We performed electrophoresis mobility shift assay (EMSA) using the *FTL* sequence bearing a TFCP2 binding motif. We observed that the fold excess unlabeled WT-TF-(wild type-TFCP2) *FTL* competitors disrupted the DNA-protein complexes in a dose-dependent manner. In contrast, an excess of unlabeled mutated (Mut) probe bearing the disrupted TFCP2 motif was unable to compete for the complex (Figure 3F, left two images). To determine whether YAP was involved in the formation of these complexes, antibodies were added simultaneously. We observed that non-specific control-IgG had no competing effects, whereas the addition of anti-YAP abrogated the DNA-protein complexes in a dose-dependent manner (Figure 3F, right two images). YAP overexpression increased, while YAP knockdown or knockout decreased, the intensity of observed DNA-protein complexes. Simultaneously, overexpressing TFCP2 and YAP further enhanced the formation of DNA-protein complexes, compared to overexpressing YAP alone, while TEADs did not have a similar synergistic role. The formation of DNA-protein complexes was not affected by HNF4a overexpression or c-Jun knockdown compared to control treatment (Figure 3G). Collectively, these findings demonstrated that YAP acts as a cotranscription factor to promote TFCP2-mediated *FTL* transcription.

### Forkhead Box A1 Stimulated by Yes-Associated Protein and Transcription Factor CP2 Is Also Responsible for *FTL* Transcription

Single knockout of YAP or TFCP2 showed similar effects on *FTL* promotor activity, and both YAP and TFCP2 knockout exhibited an additional effect compared to single knockout (Figure 2H), which suggested that TFCP2 might not be the only transcription factor acting at this site. In our previous study, we identified 5 transcription factors (including POU class 3 homeobox 2 (POU3F2), musculin (MSC), nuclear receptor subfamily 1 group D member 1 (NR1D1), FOXA1 and FOXC1) that are stimulated by both YAP and TFCP2 (Zhang et al., 2017b). Through the prediction of FIMO software, we found that the FOXA1 transcription factor could also be simultaneously bound at the TFCP2-bound motif (Supplementary Figure 3E), and ChIP experiments proved that both TFCP2 and YAP were involved in the binding of FOXA1 to the *FTL* promoter (Supplementary Figure 3F). Luciferase reporter assay experiments showed that the promoting effect of TFCP2 on the *FTL* promoter could be further promoted by FOXA1 and YAP, while the promoting effect of TFCP2, FOXA1 and YAP on the *FTL* promoter was lost after the key binding sequence was mutated (Supplementary Figure 3G). Moreover, we found that FOXA1 knockout could inhibit the activity of the *FTL* promoter, while simultaneous knockout of FOXA1 and TFCP2 and simultaneous knockout of YAP and TFCP2 inhibited *FTL* promoter activity to a similar degree (Supplementary Figure 3H). These results indicate that FOXA1, a transcription factor coregulated by YAP and TFCP2, is also involved in the regulation of the *FTL* promoter and that TFCP2 and FOXA1, as transcription factors, bind to almost the same position of the *FTL* promoter.

### Ferritin Light Chain Knockdown Abrogates Sustained Yes-Associated Protein Induced Ferroptosis Inhibition

**FIGURE 4:**
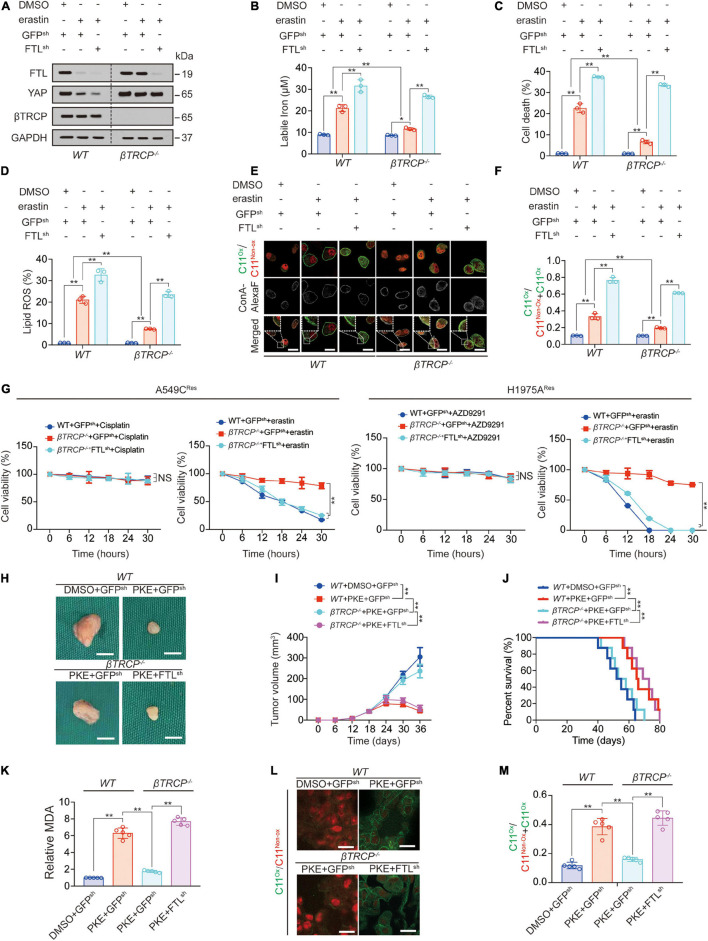
Ferroptosis inhibition induced by YAP-sustain is abrogated by FTL knockdown. **(A)** FTL, YAP and βTRCP expressions were measured in *WT* or β*TRCP^–/–^* PC9 cells with or without FTL knockdown followed by erastin (10 μM, 24 h) treatment. **(B–D)** Labile iron **(B)**, cell death **(C)** and lipid ROS generation **(D)** were measured in PC9 cells with the same treatment as that in panel A. But the treating time was 24 or 16 h. **(E,F)** Lipid peroxidation was probed by C11-BODIPY^581/591^ in PC9 cells with the same treatment as that in panel A **(E)**. The fractions of oxidized C11-BODIPY^581/591^ were calculated in each group **(F)**. Scale bar, 50 μm. **(G)** Relative cell viability was measured in WT or βTRCP^–/–^ A549 Cisplatin resistant (C^*Res*^) or H1975 AZD9291 resistant (A^*Res*^) with or without FTL knocked down and their parental cells treating with or without Cisplatin (10 μM), AZD9291 (10 μM) or erastin (10 μM) for indicated hours. **(H,I)** Xenografts that formed by H1975 cells with or without knocking down FTL. DMSO or PKE (5 mg/kg) was administrated once every day for 14 days began at day 18 after inoculation (*n* = 5/group). Represented images were shown in panel H, scale bar, 5 mm; and tumor volume was monitored every 6 days and graphed in panel I. **(J)** Survival of mice bearing xenograft with the same treatment as that in panel H (*n* = 8/group). **(K)** MDA of xenograft that formed at the endpoint of the experiments with the same treatment as that in panel H (*n* = 5/group). **(L,M)** Lipid peroxidation was probed by C11-BODIPY^581/591^ in xenograft that formed at the endpoint of the experiments with the same treatment as that in panel H (*n* = 5/group) and the representative images were shown in panel L, scale bar, 50 μm; and the fractions of oxidized C11-BODIPY^581/591^ were calculated in each group **(M)**. The data are shown as the mean ± SD from three biological replicates (including IB). *P < 0.05, **P < 0.01 indicates statistical significance. Data in **(B–D,F,K,M)** were analyzed using a one-way ANOVA test. Data in G, I were analyzed using a two-way ANOVA test. Data in **(J)** were analyzed using log rank tests.

βTRCP-knockout-induced sustained YAP leads to ferroptosis inhibition (Zhang et al., 2021). Subsequently, we investigated whether sustained YAP induces ferroptosis inhibition *via* FTL. We found that FTL knockdown further promoted erastin-induced labile iron elevation, cell death, lipid ROS generation and lipid peroxidation on the plasma membrane (Figures 4A–F); moreover, FTL knockdown abrogated βTRCP-knockout-induced inhibition of these ferroptotic effects caused by erastin treatment (Figures 4A–F). In cisplatin-resistant A549 (A549C^Res^) cells and AZD9291-resistant H1975 (H1975A^Res^) cells, we observed that erastin treatment significantly inhibited cell viability, whereas ßTRCP reversed the effects of erastin. However, FTL knockdown restored cell sensitivity to erastin, suggesting that inducing cell ferroptosis could be an alternative therapy for patients with chemotherapy or targeted therapy resistance (Figure 4G). In xenograft models, we used PKE, an *in vivo* stable erastin derivative, to replace erastin to induce ferroptosis *in vivo*. We found that βTRCP knockout partially reversed the PKE-induced decrease in tumor volume, and prolonged survival, malondialdehyde (MDA) production and lipid peroxidation (Figures 4H–M). Similar to the *in vitro* experiments, FTL knockdown abrogated βTRCP-knockout-induced inhibition of these ferroptotic effects (Figures 4H–M). Additionally, we found that the iron chelators deferoxamine (DFO) and ciclopirox olamine (CPX) reversed the YAP-and TFCP2-knockdown-induced increase in labile iron and lipid ROS generation (Supplementary Figures 4A,B). The above findings implied that FTL knockdown can reactivate ferroptosis, even in a sustained YAP situation caused by βTRCP knockout.

### Ferritin Light Chain and Ferritin Heavy Chain Are Differentially Regulated After Erastin Treatment

**FIGURE 5:**
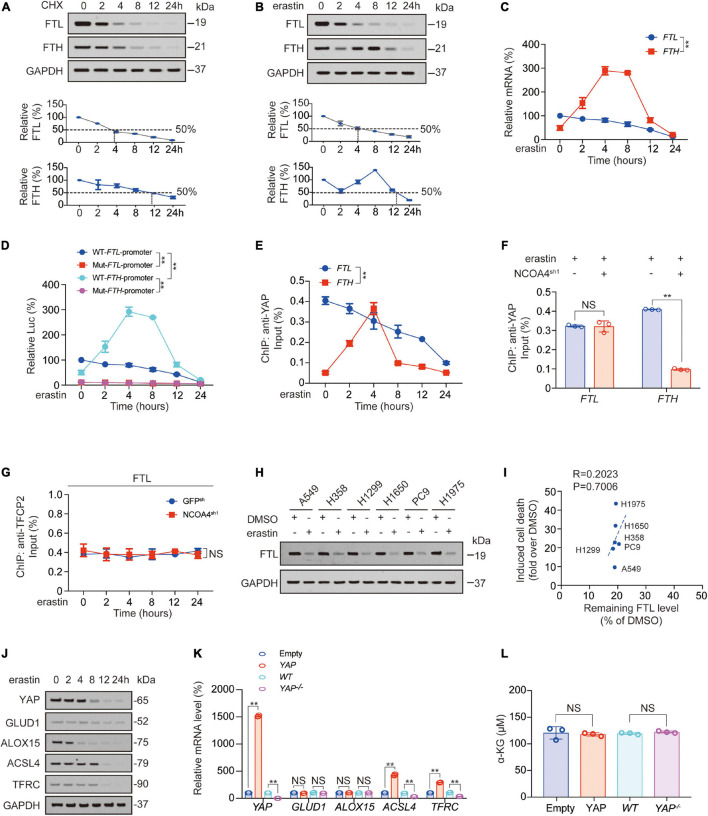
FTL and FTH were differentially regulated after erastin treatment. **(A,B)** FTL and FTH were measured by IB in PC9 cells treated with CHX (10 μg/ml, **A**) or erastin (10 μM, **B**) for indicated hours. The relative protein levels of FTL and FTH were shown as the ratios to GAPDH, and the “0h” points were arbitrarily set to 100%. **(C)** mRNA level of *FTH* and *FTL* in PC9 cells treated with erastin (10 μM) for indicated hours. **(D)** WT or TFCP2 (FOXA1)-binding motif mutant (Mut) *FTL*/*FTH* promoter Luc activity were measured in PC9 cells treated with erastin (10 μM) for indicated hours. **(E)** Enrichment of YAP at TFCP2 (FOXA1)-binding motif within the *FTL* or *FTH* promoter in PC9 cells was measured by ChIP-qPCR after erastin treatment (10 μM) for indicated hours. **(F)** Enrichment of YAP at TFCP2 (FOXA1)-binding motif within the *FTL* or *FTH* promoter in erastin (10 μM, 4 h) treated PC9 cells with or without NCOA4 knockdown was measured by ChIP-qPCR. **(G)** Enrichment of TFCP2 at TFCP2 (FOXA1)-binding motif within the *FTL* promoter in PC9 cells with or without NCOA4 knockdown was measured by ChIP-qPCR after erastin treatment (10 μM) for indicated hours. **(H)** FTL was measured by IB in indicated LUAD cells with or without erastin (10 μM, 24 h) treatment. **(I)** Correlation between induced cell death and remaining FTL level after erastin (10 μM, 24 h) treatment for 24 h. Induced cell death and remaining FTL level was calculated as fold or percentage to the ones treated with DMSO. **(J)** YAP, GLUD1, ALOX15, ACSL4 and TFRC expression were measured using IB in PC9 cells with erastin (10 μM) treatment for indicated hours. **(K,L)** *YAP, GLUD1, ALOX15, ACSL4* and *TFRC* mRNA level **(K)** and α-KG concentration **(L)** were measured in PC9 cells with YAP overexpressed or knocked down. The data are shown as the mean ± SD from three biological replicates (including IB). **P < 0.01 indicates statistical significance. Data in **(C–E,G)** were analyzed using a two-way ANOVA test. Data in **(F,K,L)** were analyzed using student’s *t*-test. Data in I were analyzed using Spearman rank-correlation analysis.

In our previous study, we reported that *FTH* transcription first increased and then decreased after erastin treatment (Zhang et al., 2021). So how did FTL change after erastin was added? We observed that compared to FTH, FTL protein was more unstable in PC9 cells. The half-life of FTH was approximately 12 h, while that of FTL was approximately 4 h (Figure 5A). Unlike FTH protein, which exhibited a decline followed by an increase and then a decline again, FTL protein continued to decline after the addition of erastin (Figure 5B). As for mRNA level, promoter activity, and YAP recruitment around the TFCP2 (FOXA1) motif, those of *FTL* were consistently decreased, while those of *FTH* increased first and then decreased (Figures 5C–E). Erastin-induced YAP recruitment to *FTH* promoter is NCOA4-dependent (Figure 5F; Zhang et al., 2021). In contrast, we found that YAP recruitment to the *FTL* promoter was not related to NCOA4 (Figure 5F). Furthermore, TFCP2 recruitment to the *FTL* promoter did not decrease during ferroptosis occurrence, nor did it change due to NCOA4 knockdown (Figure 5G). We observed that after the addition of erastin, FTL decreased to similar degrees in different LUAD cell lines, and the remaining FTL level was not correlated with the degree of ferroptosis, suggesting that although FTL could affect ferroptosis sensitivity, it was not the cause of the differences in ferroptosis sensitivity among LUAD cell lines (Figures 5H,I).

In addition, we observed that FTL overexpression slightly inhibited FTH expression, whereas FTL knockdown promoted FTH expression (Supplementary Figures 5A,B). However, DFO treatment reversed this FTL knockdown-induced effect, and we also found that the change in the trend of FTH was similar to that of labile iron, suggesting that FTL might be regulated by labile iron (Supplementary Figures 5A,C). A previous study reported that an increase in labile iron could promote the binding of YAP to the *FTH* promoter to stimulate *FTL* transcription, therefore supporting our hypothesis (Zhang et al., 2021).

We also tested whether acyl-CoA-synthetase long chain family member 4 (ACSL4); transferrin receptor (TFRC); glutamate dehydrogenase 1 (GLUD1, which catalyzes the oxidative deamination of glutamate to α-KG; and a key enzyme in 4-HNE synthesis, arachidonate 15-lipoxygenase (ALOX15), were also influenced by YAP after erastin treatment. We found that the half-life of YAP was between 4 and 8 h; the half-life of ACSL4 and TFRC was longer than that of YAP, approximately 8–12 h; the half-life of ALOX15 was shorter than that of YAP, approximately 2–4 h; and the expression of GLUD1 was almost unchanged within 24 h. These findings indicated that ACSL4 and TFRC, with longer half-lives than YAP, might be regulated by YAP, whereas ALOX15, with a shorter half-life than YAP, or GLUD1, with a half-life longer than 24 h, were not regulated by YAP (Figure 5J and Supplementary Figure 5D). We also found that *ACSL4* and *TFRC* mRNA levels were positively controlled by YAP, whereas *ALOX15* and *GLUD1* mRNA and a-KG levels were not regulated by YAP (Figures 5K,L). Therefore, the decrease in YAP caused by erastin treatment was followed later by a decrease in ACSL4 and TFRC.

### KAT5-Induced Acetylation of FTL Is Decreased During Ferroptosis

**FIGURE 6:**
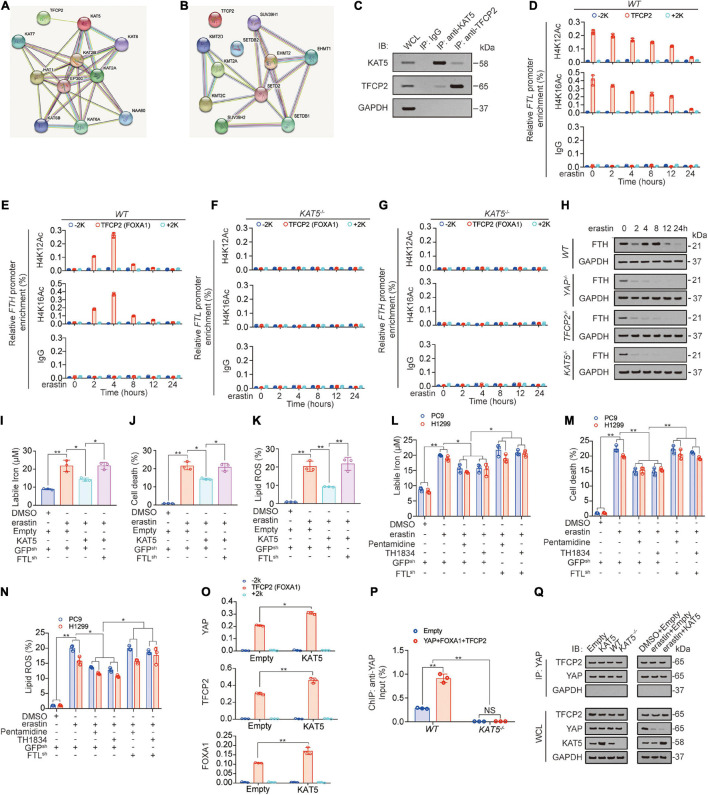
KAT5-induced *FTL* acetylation suppresses ferroptosis. **(A,B)** STRING analysis revealed TFCP2 interacted acyltransferase **(A)** and methyltransferases **(B)** (confidence = 0.4). **(C)** Co-immunoprecipitation experiments performed in PC9 cells using IgG, anti-KAT5 and anti-TFCP2 antibodies, and further analysis of KAT5, TFCP2 and GAPDH expression by IB. **(D–G)** The enrichments of H4K12Ac and H4K16Ac at –2k, TFCP2 (FOXA1) motif or 2k regions of *FTL* **(D,F)** or *FTH* **(E,G)** promoter were calculated as the percentage of input chromosomal DNA *via* ChIP using the corresponding antibodies in *WT* **(D,E)** or *KAT5^–/–^* (F, G) PC9 cells with erastin (10 μM) treated for indicated times. IgG was used as the parallel control. **(H)** FTH expression were measured using IB in *WT, YAP^–/–^, TFCP2^–/–^* and *KAT5^–/–^* PC9 cells with erastin (10 μM) treatment for indicated hours. **(I–K)** Labile iron, cell death and lipid ROS were measured in PC9 cells with or without KAT5 overexpression combined with FTL knockdown before erastin (10 μM, 24 h or 16 h) treatment. **(L,M)** Labile iron, cell death and lipid ROS generation were measured in PC9 and H1299 cells with or without FTL knocked down. Pentamidine (30 μM) and TH1834 (100 μM) were used to pretreat cells for 8 h before further treated with erastin (10 μM, 24 h). **(N)** The enrichments of YAP, TFCP2 and FOXA1 at –2k, TFCP2 (FOXA1) motif or 2k regions of *FTL* promoter were calculated as the percentage of input chromosomal DNA *via* ChIP using the corresponding antibodies in PC9 cells with or without KAT5 overexpressed. **(O)** Enrichment of YAP at TFCP2 (FOXA1)-binding motif within the *FTL* promoter in *WT* or *KAT5^–/–^* PC9 cells with or without YAP, TFCP2 and FOXA1 overexpressed was measured by ChIP-qPCR. **(P)** Co-IP experiments were performed using anti-YAP in PC9 cells with KAT5 overexpression or knockdown with or without erastin treatment (10 μM, 24 h). The YAP level in each co-IP samples was adjusted to the same protein content. The indicated proteins in co-IP samples or WCL were measured by IB. The data are shown as the mean ± SD from three biological replicates (including IB). *P < 0.05, **P < 0.01 indicates statistical significance. Data in **(I–M,O)** were analyzed using a one-way ANOVA test. Data in **(N)** were analyzed using student’s *t*-test.

Histone modification, especially methylation and acetylation, is critical for transcription (Tessarz and Kouzarides, 2014; Bach et al., 2015). We further investigated whether methyltransferase or acetyltransferase played important roles in *FTL* transcription. After a STRING analysis detecting potential TFCP2-binding methyltransferase/acetyltransferase, we found that KAT5 had the highest possibility of binding with TFCP2 (Figure 6A). Co-IP experiments confirmed that TFCP2 interacted with KAT5 (Figure 6B). The hallmarks of open chromatin related to KAT5 are acetyl-histone H4 lysine 12 (H4K12Ac) and acetyl-histone H4 lysine 16 (H4K16Ac) (Li and Wang, 2017; Uchida and Shumyatsky, 2018). We found that both H4K12Ac and H4K16Ac levels in the TFCP2 (FOXA1) motif of the *FTL* promoter continuously decreased after erastin treatment (Figure 6D);, whereas in the TFCP2 (FOXA1) motif of the *FTH* promoter, H4K12Ac and H4K16Ac levels exhibited an upward trend followed by a downward trend (Figure 6E), and these effects were KAT5-dependent (Figures 6F,G, KAT5 knockout efficiency was validated in Figure 6P). We found that the knockout of YAP, TFCP2 or KAT5 interrupted the increase in FTH at 2 h after erastin addition, which suggested that YAP, TFCP2 and KAT5 could also control the transcription of FTH (Figure 6H). Interestingly, ectopically expressed KAT5 partially reversed the erastin-induced elevation of labile iron, cell death and lipid ROS generation; however, the reversal effect was abolished when FTL was further knocked down (Figures 6I–K, KAT5 overexpression efficiency was validated in Supplementary Figure 6I). In addition, we observed that two KAT5 inhibitors, pentamidine and TH1834 (Brown et al., 2016), could enhance ferroptosis resistance by partially reversing erastin-induced elevation of labile iron, cell death and lipid ROS production. However, these effects were abolished when FTL was further knocked down (Figures 6L–N). We further investigated the role of KAT5 in binding between transcription (co-) factors and the *FTL* promoter, and found that the overexpression of KAT5 promoted the enrichment of YAP, TFCP2, and FOXA1 near the TFCP2 (FOXA1) motif (Figure 6N). However, after KAT5 knockout, YAP enrichment near the TFCP2 (FOXA1) motif was abolished and could not be promoted by YAP, TFCP2, and FOXA1 overexpression (Figure 6O). We also found that KAT5 could not affect the binding of the TFCP2 protein to YAP, even after erastin treatment (Figure 6P). Therefore, it is clear that the role of KAT5 is to promote the enrichment of YAP, TFCP2 and FOXA1 near the TFCP2 (FOXA1) motif in the *FTL* promoter. In addition, we found that the overexpression of FTH or FTL reduced labile iron levels, whereas the knockdown of FTH or FTL increased labile iron levels (Supplementary Figure 6A). However, neither FTH nor FTL influenced YAP, TFCP2, βTRCP or KAT5 expression (Supplementary Figure 6B), suggesting that FTH and FTL act downstream of YAP, TFCP2, βTRCP and KAT5 and have no role in regulating their expression. Altogether, the above data suggested that KAT5-induced acetylation of the *FTL* promoter is continuously decreased after erastin treatment, and this acetylation might play a ferroptosis-resistance role.

### FTL Transcription Also Determines Ferroptosis Sensitivity in Glioblastoma Cells

Subsequently, we also validated our model in the U87 glioblastoma cell line. We found that in U87 cells, YAP overexpression increased the enrichment of YAP and TFCP2 around the TFCP2 (FOXA1) binding motif of the *FTL* promoter, and this effect was further enhanced by simultaneously overexpressing TFCP2 but not TEADs (Supplementary Figure 6C). In addition, *FTL* mRNA levels were positively regulated by YAP and TFCP2 (Supplementary Figure 6D), suggesting that YAP and TFCP2 control *FTL* transcription in U87 cells. We also found that βTRCP knockout or KAT5 overexpression led to reduced ferroptotic sensitivity, and these effects could be abolished by FTL knockdown (Supplementary Figures 6E–L). In conclusion, *FTL* transcription is controlled by the YAP-TFCP2-dependent transcription complex and regulates ferroptosis sensitivity in glioblastoma cells.

### The Degree of Lipid Peroxidation May Be Correlated With the Degree of Lung Adenocarcinoma Malignancy

**FIGURE 7:**
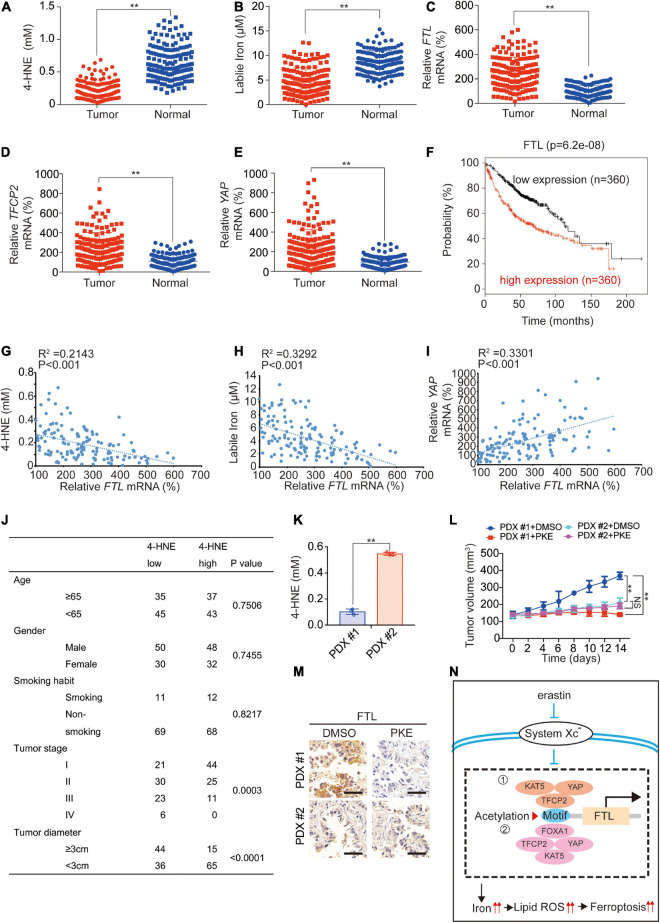
Clinical significance of the study. 4-HNE **(A)**, labile iron **(B)**, mRNA levels of *FTL* **(C)**, *TFCP2* **(D)** and *YAP* **(E)** were analyzed in LUAD tumor (*N* = 150) and adjacent normal (*N* = 150) tissues. **(F)** Kaplan–Meier survival plots of FTL were obtained from the KM plotter database. **(G–I)** Analysis of correlation between *FTL* mRNA and 4-HNE **(G)**, labile iron **(H)** as well as *YAP* mRNA **(I)**. **(J)** Association between 4-HNE level and clinicopathological parameters including age, gender, smoking habit, tumor stage and tumor diameter. **(K)** 4-HNE level in PDX#1 and PDX#2 before PKE treatment. **(L)** Volume of PDX for indicated times after treating with or without PKE (5 mg/kg) once every day for 14 days (*n* = 8/group). **(M)** FTL expression was measured in PDX model with or without PKE (5 mg/kg) treatment at day 14 by IHC. Scale bar, 200 μm. **(N)** The model of the study. When system Xc^–^ is inhibited by erastin, the acetylation induced by YAP-TFCP2 dependent transcription (➀ TFCP2 as the transcription factor; ➁ YAP and TFCP2 co-regulated FOXA1 as the transcription factor) of *FTL* promoter is inhibited, which suppressed the transcription of *FTL*. This further leads to a labile iron and ferroptosis sensitivity elevation in LUAD cells. The data are shown as the mean ± SD from three biological replicates. **P < 0.01 indicates statistical significance. Data in A-E, K were analyzed using student’s *t*-test. Data in (G-I) were analyzed using Spearman rank-correlation analysis. Data in **(L)** were analyzed using a two-way ANOVA test.

We further investigated the clinical association of the above factors. In fresh tissues, we found that 4-HNE and labile iron were downregulated, while *FTL, TFCP2* and *YAP* mRNA levels were upregulated in LUAD tissues compared to adjacent normal tissues (Figures 7A–E and Supplementary Figure 5). High expression of FTL was significantly associated with poor prognosis (Figure 7F), and *FTL* mRNA levels were negatively correlated with 4-HNE and labile iron and positively correlated with *YAP* mRNA levels (Figures 7G–I), demonstrating that FTL expression increased in LUAD and was negatively related to the degree of lipid peroxidation.

Subsequently, we analyzed the association between lipid peroxidation and LUAD progression. We found that a low 4-HNE level was negatively associated with tumor stage (*P* = 0.0003) and tumor diameter (*P* < 0.0001) but was not related to age, sex or smoking habit (Figure 7J). In addition, we observed that in tumors with low 4-HNE levels, YAP, TFCP2, FTL expression and YAP enrichment in the *FTL* promoter were significantly higher than those in adjacent normal tissues, whereas these phenomena were not obvious in tumors with high 4-HNE levels (Supplementary Figures 6A–D). We constructed two patient-derived xenograft (PDX) models with 4-HNE low (PDX #1) and 4-HNE high (PDX #2) (Figure 7K) and found that PKE inhibited tumor growth and FTL levels in the PDX #1 model to a greater extent than it did in the PDX #2 models (Figures 7L,M). Additionally, we cultured 6 primary cells from LUAD patients, of which #1-#3 were 4-HNE low cells and #4-#6 were 4-HNE high cells. We found that the 4-HNE level in cells #1-#3 increased by an average of 10.00 times 24 h after the addition of erastin, which was significantly higher than that in cells #4-#6 (1.50 times) (Supplementary Figure 7F). In addition, the increasing trend in labile iron and cell death and the decreasing trend in FTL after erastin treatment in cells #1-#3 were also significantly larger than those in cells #4-#6 (Supplementary Figures 7G–J), which further indicated that cells with lower 4-HNE levels were more sensitive to ferroptosis. These findings implied that LUAD with low lipid peroxidation levels could be associated with high ferroptosis sensitivity.

## Discussion

Yes-associated protein regulation of ferroptosis has two sides. In cells, especially those with metastatic characteristics, E-cadherin inhibition leads to YAP activation, which further activates ferroptosis stimulators ACSL4 and TFRC, but it also converts labile iron into ferritin-bound iron to inhibit endogenous ferroptosis by promoting the transcription of *FTL*, assisting cells in storing a large amount of iron and resulting in a highly sensitive environment for ferroptosis (Wu et al., 2019; Zhang et al., 2021). When ferroptotic stimuli such as erastin are added, YAP decline causes unsustainable *FTH* and *FTL* transcription (Zhang et al., 2021). Furthermore, under the support of other reactions, such as ferritinophagy, a large amount of ferritin-bound iron is converted into labile iron (Mancias et al., 2014). However, the decrease in ACSL4 and TFRC in this process might restrict the outbreak of ferroptosis to a greater extent, which might be a mechanism of cell self-protection, indicating that YAP might balance ferroptosis in cells.

A previous study reported that TFCP2 is a key cofactor of YAP in regulating transcription. TFCP2 can recruit other YAP-dependent transcription factors, including TEAD, FOXA1, FOXC1, MSC, NR1D1, and POU3F2, and activate these transcription factors (Zhang et al., 2017b). In addition, TFCP2 acts directly as a YAP-regulated transcription factor that mediates transcription of *FTH* (Zhang et al., 2021). Here, we found that TFCP2 can simultaneously act as a transcription factor regulated by YAP and together with YAP to regulate FOXA1-induced transcription of *FTL*, and the binding sites of FOXA1 and TFCP2 on the *FTL* promoter partially overlap, suggesting that their regulation of FTL may be a coexisting and competitive regulation mode. However, whether there are other transcription factors regulated by YAP or TFCP2 involved in the transcriptional regulation of *FTL* near this site still needs to be studied.

Here, we found that tumors with lower 4-HNE levels may have higher ferroptosis sensitivity. Compared to high-4-HNE tumors, the FTL level in low-4-HNE tumors was generally higher, and FTL decreases more strongly after inhibition by system Xc^–^, producing more labile iron, resulting in a more obvious increase in 4-HNE and ferroptotic cell death. However, tumors with high 4-HNE did not produce a significant increase in labile iron or 4-HNE and a decrease in FTL after inhibition by system Xc^–^, and therefore did not cause significant ferroptosis. In addition, 4-HNE is relatively low in high-stage tumors compared to low-stage tumors and normal tissues. Consistent with previous studies, ferroptosis is generally more sensitive in tumors with a higher degree of malignancy, such as tumors with a high metastatic tendency, advanced stage, and drug resistance (Viswanathan et al., 2017; Wu et al., 2019; Elgendy et al., 2020; Zhang et al., 2021). This might be because many targets of ferroptosis (including SLC7A11, YAP, and ADCY10) are positively correlated with tumor progression, and they guarantee the outbreak of cell ferroptosis after receiving an external ferroptosis-promoting signal (Wu et al., 2019; Ma et al., 2021; Zhang et al., 2021). Because ferroptotic therapy specifically targets malignant cells and protects normal cells, it has great potential in clinical applications.

This study and existing studies have shown that ferroptosis may have a good therapeutic effect on patients with resistance to drugs such as BRAF inhibitors, cisplatin, icotinib, and osimertinib (Friedmann Angeli et al., 2019; Fu et al., 2021; Zhang et al., 2021). Therefore, we believe that in future clinical treatment, using ferroptotic therapy to treat drug-resistant patients or using ferroptotic therapy directly in combination with chemotherapy or targeted drugs can effectively avoid tumor progression caused by drug resistance. In addition, several ferroptotic sensitivity biomarkers (such as ACSL4, calcium/calmodulin-dependent protein kinase kinase 2 (CAMKK2) and ADCY10 (Doll et al., 2017; Wang et al., 2021; Zhang et al., 2021)) and therapeutic targets (such as FTL and YAP (Wu et al., 2019; Zhang et al., 2021)) have been identified, all of which provide support for ferroptosis use in clinical oncology therapy. Of course, although ferroptotic-promoting drugs such as sorafenib have been applied in clinical tumor treatment (Chen et al., 2021), the extensive application of ferroptotic therapy in clinical studies requires the guidance of clinical studies with large sample sizes and multicenter and finely grouped specimens. In conclusion, we found a mechanism through which the inhibition of system Xc^–^ followed by further inhibition of YAP-TFCP2-KAT5 complex-mediated *FTL* transcription induces ferroptosis (Figure 7N). This mechanism may provide a therapeutic basis for ferroptotic therapy in tumor patients with low 4-HNE.

## Materials and Methods

### Cell Culture and Vectors

H358, H1650, PC9, H1975, A549 and H1299 cell lines were purchased from Shanghai Fuheng Biotechnology Co., Ltd and cultured in DMEM. A549C^Res^ and H1975A^Res^ cells were acquired from our previous study (Zhang et al., 2021). Cells were treated with erastin (Selleckchem, Houston, TX, United States), cycloheximide (CHX, Sigma, St Louis, MO, United States), cisplatin (MedChemExpress, Monmouth Junction, NJ, United States), AZD9291 (MedChemExpress), pentamidine (MedChemExpress), TH1834 (Sigma), Dox (Clontech, Mountain View, CA, United States), EGCG (MedChemExpress), EGTA (MedChemExpress), H89 (MedChemExpress), Rp-cAMPs (MedChemExpress), glucose (Sigma), GlcNAc (Sigma), GlcN (MedChemExpress), DFO (Selleckchem), or CPX (Selleckchem). Patient-derived primary LUAD cells were established from LUAD tissues as previously described. Briefly, tissue samples less than 1.0 cm^3^ in size without necrosis were immediately washed with ice-cold Dulbecco’s phosphate buffered saline (DPBS) 3 times before resuspension in Dulbecco’s modified Eagle medium (DMEM) containing collagenase I (2 mg/ml, Solarbio, Shanghai, China) at 37°C for 4 h. After washing 3 additional times with DMEM, cells were cultured under routine conditions (Zhang et al., 2021). The YAP^HA^, YAP^sh1^, YAP^sh2^, TFCP2^Myc^, YAP^Del–WW1–HA^, YAP^Del–WW2–HA^, YAP^Del–2WW–HA^, TFCP2^Y269A–Myc^, TFCP2^sh1^, TFCP2^sh2^, c-Jun, HNF4a, Runx2, STAT3, c-Jun^sh1^, NCOA4^sh^, FOXA1, XBP1s plasmids and sgRNA targeting βTRCP and ADCY10 were acquired from our previous studies (Wang J. et al., 2015; Qiao et al., 2016; Zhang et al., 2017b, 2018, 2019, 2021). The FTL^sh^ and FTH^sh^ plasmids were constructed using PLKO.1. FTH-, FTL-, KAT5-, P73- and SMAD2-expressing plasmids were purchased from Biolink (Shanghai, China). sgRNAs targeting YAP (YAP^KO1^ YAP^KO2^), TFCP2 (TFCP2^KO1^ TFCP2^KO2^) KAT5 and FOXA1 were constructed using lentiCRISPR v2. sgRNA targeting βTRCP was acquired from a previous study (Zhang et al., 2021). Related promoter regions of human *FTH* and *FTL* genes were PCR amplified from gDNA of PC9 cells and cloned into pGL4.21 (Promega, Madison, WI, United States) vectors. The primers and gRNA are summarized in Supplementary Table 1.

### Lentivirus Package

On day 1, 293T cells were plated in a 10 cm culture dish at a density of 10 × 10^6^ and cultivated with 10 ml DMEM. On day 2, 1 ml culture medium (serum-free and antibiotic-free) was taken and placed in an EP tube; 45 μl transfection reagent A, 7.5 μg pspAX and pmD2G mixed plasmid B (reagent A and plasmid B were bought from Biolink LTD., Shanghai, China), and 7.5 μg target plasmid was added; and the mixed reagent was manually inverted and incubated for 30 min at room temperature. The above mixture was slowly added to the 293T cells, and then the mixture was placed in an incubator at 37°C and 5% CO_2_ for 24 h. At day 3, the viral supernatant was collected and centrifuged for 10 min (3000 rpm/min, 4°C), the supernatant was filtered with a 0.45 μm filter, and then the target cell was transfected with the filtered virus supernatant directly or the supernatant was stored at −80°C.

### Mouse Experiments and Tissue Samples

For xenograft experiments, six-week-old athymic nude mice were purchased from Shanghai Jiesijie Experimental Animal Co., Ltd., and *WT* or β*TRCP^– /–^* PC9 cells with or without FTL knockdown were subcutaneously injected into each mouse. Dimethyl sulfoxide (DMSO) or PKE (5 mg/kg, MedChemExpress) was subcutaneously injected once every day after xenografts were obviously formed. For the generation of PDX mouse models, fresh LUAD tissues 2–3 mm^3^ in size were subcutaneously implanted into six-week-old athymic nude mice. After successful passage, the PDX mice were used for further study. The tumor volume was calculated as 0.5 x L x W^2^, with L indicating length and W indicating width. All mouse experiments were performed according to the institutional guidelines of Shanghai Chest Hospital. Tumorous and adjacent lung tissues of patients (mean age ± SD, 63.44 ± 10.22 years; male:female ratio, 1.14:1) were acquired from the Shanghai Chest Hospital from September 2013 to October 2019 under institutional approval. Informed written consent was obtained from all patients.

### Immunofluorescence, Immunoblotting, and Immunohistochemistry

IF, IB and IHC were performed according to conventional protocols. For IF, the fluorescent probe C11-BODIPY^581/591^ (Invitrogen, Carlsbad, CA, United States) was used to directly measure oxidized lipids and conA-AlexaF (Invitrogen) was employed to visualize the membrane. Images were captured at emission at 580/600 nm (the non-oxidized form, red) and 490/510 nm (the oxidized form, green) and then merged to demonstrate the fraction of oxidized C11-BODIPY^581/591^.

For IB, the primary antibodies included anti-YAP (Abcam, #ab52771 or Santa Cruz Biotechnology, Santa Cruz, CA, United States, #sc-101199), anti-GAPDH (CST, Boston, MA, United States, #5174), anti-TFCP2 (CST, #80784), anti-LATS (Abcam, #ab70561), anti-SMAD2 (Abcam, #ab33875), anti-TRIB2 (Abcam, #ab84683), anti-CREB (Abcam, #ab32515), anti-HA (CST, #2367), anti-Myc (CST, #2276), anti-FTL (Abcam, #ab69090), anti-HSPB1 (Abcam, #ab5579), anti-CISD1 (Abcam, ab133970), anti-βTrCP (Abcam, #ab71753), anti-FTH (Abcam, #ab65080), anti-KAT5 (Abcam, #ab172415), anti-GLUD1 (Abcam, #ab153973), anti-ALOX15 (Abcam, ab242062), anti-ACSL4 (Abcam, ab155282) and anti-TFRC (Abcam, ab214039).

For IHC, the primary antibodies included was anti-YAP (Abcam, #ab52771), anti-TFCP2 (CST, #80784) and anti-FTL (Abcam, #ab69090). IHC scores were computed by multiplying the staining intensity grade (0, 1, 2, and 3 represented negative, weak-positive, moderate-positive and strong-positive, respectively) by the positive rate score (0, 1, 2, 3 and 4 represented positive areas of ≤_5%, 6–25%, 26–50%, 51–75%, and ≥_76%, respectively) as we previously described (Ma et al., 2021).

### Luciferase Reporter Assay

Luciferase reporter vectors were cotransfected with a Renilla reporter plasmid into indicated cells, and the luciferase activities were measured using dual-luciferase reagent (Promega).

### Measurements of Cell Viability, Cell Death, and Lipid Reactive Oxygen Species

Cell viability was measured using a CellTiter-Glo luminescent cell viability assay (Promega). Cell death was analyzed by staining with SYTOX Green (Invitrogen) before flow cytometry analysis. Lipid ROS was measured by adding C11-BODIPY (Invitrogen) to a final concentration of 1.5 μM for 20 min before cell harvest. Lipid ROS-positive cells were subsequently assessed by a flow cytometer.

### Coimmunoprecipitation

After whole cell lysate (WCL) samples were acquired, cell lysates were incubated with protein A/G magnetic beads (Novex, Oslo, Norway) and western/IP lysis buffer (Beyotime, Haimen, China). Immunoprecipitates were washed five times. WCL samples were first subjected to IB analysis. The target proteins bound by the antibody were adjusted to the same content in each co-IP sample according to the gray value. Then, the co-IP samples were subjected to IB analysis to investigate the binding amount of other proteins to the target protein. The antibodies used for co-IP were anti-Myc (CST, #2276 or #2278), anti-HA (CST, #3724 or #2367), anti-YAP (Abcam, #ab52771 or Santa Cruz, #sc-101199), anti-TFCP2 (CST, #80784) and anti-KAT5 (Abcam, #ab137518).

### Protein Ligation Assay Experiments

Protein ligation assay was performed to identify the direct interactions between two proteins using the Duolink *in situ* Red Starter Kit (mouse/rabbit) (Sigma-Aldrich, St. Louis, MO, United States). The detailed procedure was described in a previous study (Zhang et al., 2017b). The antibodies used were anti-Myc (CST, #2276) and anti-HA (CST, #3724).

### Metabolite Assay

Labile iron, 4-HNE, α-KG and MDA were measured using kits from Abcam. GSH and phospholipids were measured using the kits from Sigma.

### Real-Time Quantitative RT-PCR (qPCR)

Total RNA was extracted using TRIzol (Ambion, Carlsbad, CA, United States) and reverse-transcribed into complementary DNA using the PrimeScript^TM^ RT reagent Kit (Perfect Real Time) (Takara, Dalian, China). Quantitative PCR was performed using the SYBR premix Ex Taq (Takara) kit. For the evaluation of XBP1 splicing, semi-qPCR was performed. PCR was terminated at cycle 29 and the products were visualized by agarose gel electrophoresis. The primers are listed in Supplementary Table 1.

### Chromatin Immunoprecipitation and Re-Chromatin Immunoprecipitation

ChIP and Re-ChIP experiments were performed using kits from Active Motif (Carlsbad, CA, United States).

For ChIP experiments. Cells (2 × 10^7^) were fixed using 1% formaldehyde, washed with PBS and lysed using lysis buffer. After sonication, protein-DNA complexes were incubated overnight with antibody-coupled protein G beads at 4°C. On the 2nd day, DNA was eluted in 1% SDS/0.1 M NaHCO_3_, reversed cross-linked at 65°C, purified *via* phenol/chloroform extraction and ethanol precipitation, and subjected to subsequent analysis.

For Re-ChIP experiments, complexes were eluted by incubation for 30 min at 37°C in 10 mM DTT. After centrifugation, the supernatant was diluted 20 times with Re-ChIP buffer (1% Triton X-100, 2 mM EDTA, 150 mM NaCl, 20 mM Tris–Hcl, pH 8.1) for further analysis.

The primary antibodies used in ChIP and Re-ChIP experiments were anti-YAP (CST, #14074), anti-TFCP2 (CST, #80784), anti-STAT3 (CST, #12640), anti-FOXA1 (Abcam, #ab170933), anti-IgG (CST, #3900), anti-H4K12Ac (CST, #13944) and anti-H4K16Ac (CST, #13534).

### Electrophoretic Mobility Shift Assay

Electrophoretic mobility shift assay was performed as described in a previous study (Chen et al., 2018). A light shift kit (Pierce, Rockford, IL, United States) was used. Nuclear extracted proteins were prepared using a kit from Active Motif and incubated in the reaction buffer with or without DNA competitors (either WT or mutant) on ice followed by the addition of biotin-labeled probes (synthesized and 5′ labeled by Sangon Inc., Shanghai, China). Antibodies against YAP (CST, #14074) or IgG (CST, #3900) were added to the mix before adding the probe. All DNA-protein complexes were resolved by electrophoresis on 5% native polyacrylamide and blotted to Immobilon-My + transfer membranes (Millipore, Billerica, MA). The probes used are listed in Supplementary Table 1.

### Bioinformatic Analysis

The TFCP2 binding motif was acquired from the JASPAR database (Fornes et al., 2020). Potential TFCP2-binding methyltransferase/acetyltransferase was detected using the STRING database (Szklarczyk et al., 2019). Kaplan–Meier survival plots of FTL were obtained from the KM plotter database (Nagy et al., 2021). The FIMO tool was used to find the binding site of transcription factors in the promoter (Grant et al., 2011).

### Statistical Analysis

The tests used to examine the differences between groups were Student’s *t*-test, one-way and two-way ANOVA and the χ^2^ test. The correlation between two groups was evaluated by Spearman rank-correlation analysis. A *P* < 0.05 was considered statistically significant.

## Data Availability Statement

The raw data supporting the conclusions of this article will be made available by the authors, without undue reservation.

## Ethics Statement

The animal study was reviewed and approved by Institutional Ethics Committee of Shanghai Chest Hospital.

## Author Contributions

YM and SQ researched, analyzed data, and wrote the manuscript. HW and JC collected and analyzed clinical samples, and researched data. XT constructed the plasmids. YM performed the mouse experiments. XZ designed and organized the pictures, designed the study, and wrote the manuscript. XX researched and analyzed data. SQ performed bioinformatics analysis and designed the study. CZ, LC, and XX researched and analyzed data, and contributed to the discussion. All authors contributed to the article and approved the submitted version.

## Conflict of Interest

The authors declare that the research was conducted in the absence of any commercial or financial relationships that could be construed as a potential conflict of interest.

## Publisher’s Note

All claims expressed in this article are solely those of the authors and do not necessarily represent those of their affiliated organizations, or those of the publisher, the editors and the reviewers. Any product that may be evaluated in this article, or claim that may be made by its manufacturer, is not guaranteed or endorsed by the publisher.

## Footnotes

1
http://meme-suite.org/doc/fimo.html

## Abbreviations

4-HNE: 4-hydroxynonenal
A549CRes: cisplatin-resistant A549
ACSL4: acyl-CoA synthetase long chain family member 4
ADCY10: adenylate cyclase 10
ALOX15: arachidonate 15-lipoxygenase
AMOT: angiomotin
CAMKK2: calcium/calmodulin-dependent protein kinase kinase 2
cAMP: cyclic adenosine monophosphate
ChIP: chromatin immunoprecipitation
CISD1: CDGSH iron sulfur domain 1
co-IP: coimmunoprecipitation
CPX: ciclopirox olamine
CREB: cAMP responsive element binding protein
Cyr61: cysteine-rich angiogenic inducer 61
DFO: deferoxamine
Dox: doxycycline
EMSA: electrophoresis mobility shift assay
EGCG: epigallocatechol gallate
EGTA: ethylenebis (oxyethylenenitrilo) tetraacetic acid
FOXA1: forkhead box A1
FPN: ferroportin
FTH: ferritin heavy chain
FTL: ferritin light chain
GFPT1: glutamine-fructose-6-phosphate transaminase
GlcN-6-P: glucosamine-6-phosphate
GlcNAc: N-acetylglucosamine
GlcNAc-6-P: N-acetylglucosamine-6-phosphate
GlcN: glucosamine
GLUD1: glutamate dehydrogenase 1
GLUT3: glucose transporter type 3
GOT1: glutamic-oxaloacetic transaminase 1
GSH: glutathione
H1975ARes: AZD9291-resistant H1975
H4K12Ac: acetyl-histone H4 lysine 12
H4K16Ac: acetyl-histone H4 lysine 16
HBP: hexosamine biosynthesis pathway
HK2: hexokinase 2
HNF4a: hepatocyte nuclear factor 4 alpha
HSPB1: heat shock protein family B member 1
IREB2: iron responsive element binding protein 2
KAT5: lysine acetyltransferase 5
LATS: large tumor suppressor kinase
LUAD: lung adenocarcinoma
MDA: malondialdehyde
MSC: musculin
NCOA4: nuclear receptor coactivator 4
NR1D1: nuclear receptor subfamily 1 group D member 1
NRF2: nuclear factor erythroid 2 like 2
NUDT9: nudix hydrolase 9
O-GlcNAcylation: O-linked beta-N-acetylglucosaminylation
P73: tumor protein 73
PFKFB3: 6-phosphofructo-2-kinase/fructose-2,6-biphosphatase 3
PHKG2: phosphorylase kinase catalytic subunit gamma 2
PKA: protein kinase A
PKE: piperazine erastin
PLA: protein ligation assay
POU3F2: POU class 3 homeobox 2
PSAT1: phosphoserine aminotransferase 1
ROS: reactive oxygen species
Runx2: RUNX family transcription factor 2
SLC5A3: solute carrier family 5 member 3
SMAD2: SMAD family member 2
STAT3: signal transducer and activator of transcription 3
STK11: serine/threonine kinase 11
TEADs: TEA domain family members
TFCP2: transcription factor CP2
TFRC: transferrin receptor
TRIB2: tribbles pseudokinase 2
VGLL4: vestigial-like family member 4
YAP: yes-associated protein
α-KG: α-ketoglutaric acid
βTRCP: beta-transducin repeat-containing homolog protein.
