# Supplementary material for: Transcriptional Repression of Ferritin Light Chain Increases Ferroptosis Sensitivity in Lung Adenocarcinoma
Source: Front Cell Dev Biol. 2021 Oct 26;9:719187. doi: 10.3389/fcell.2021.719187 (PMC8576304; doi:10.3389/fcell.2021.719187)
Supplement: Supplementary file 1 [file Data_Sheet_1.docx]

**Figure S1.**


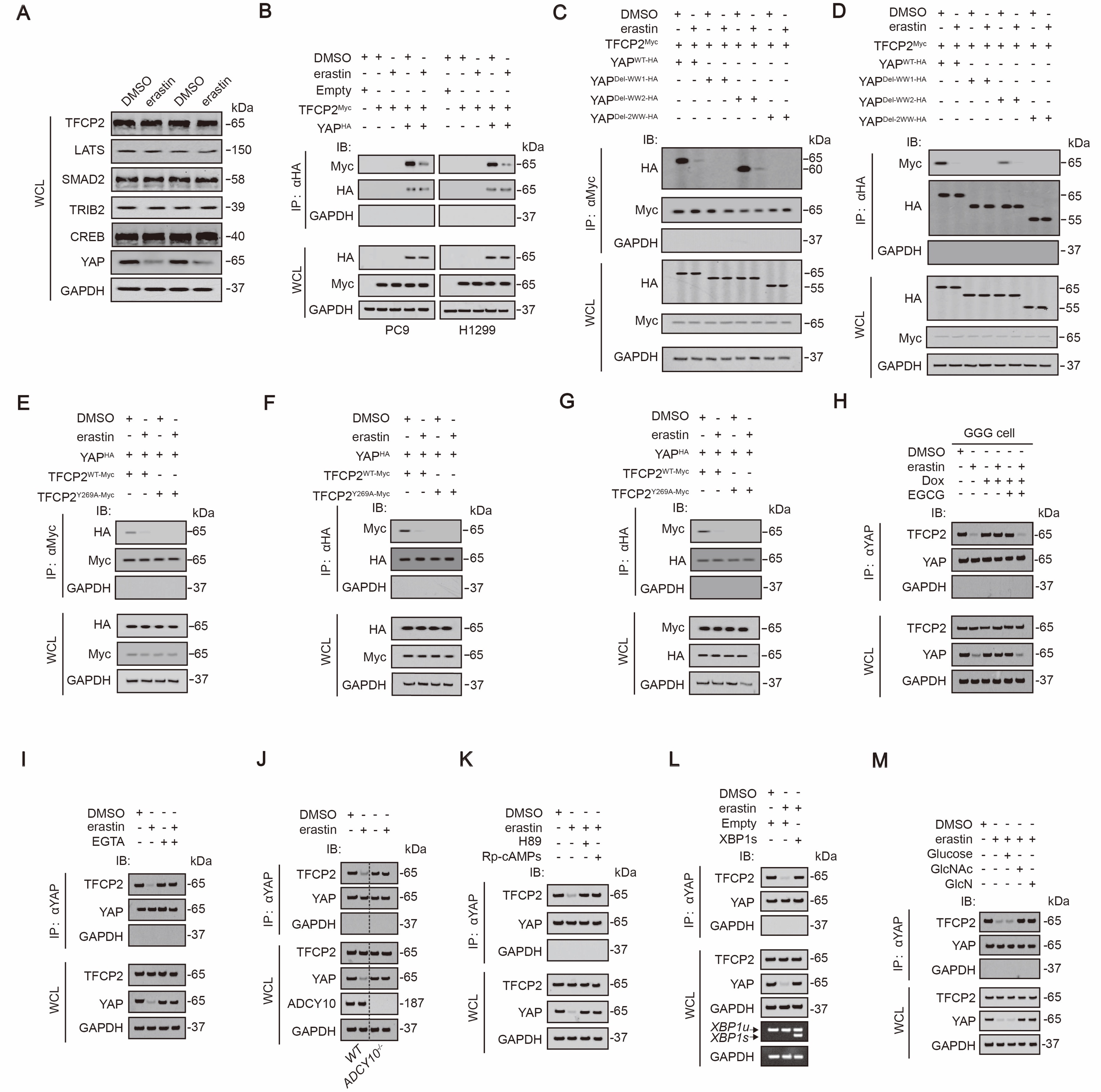


**Figure S1. Supplementary to Figure 1.**

(A) TFCP2, LATS, SMAD2, TRIB2, CREB and YAP levels in WCL of co-IP experiment in Figure 1A were measured using indicated antibodies by IB.

(B) A Co-IP experiment was performed using anti-HA antibodies in control cells and PC9 or H1299 cells with the indicated vectors transfected before erastin (5 µM, 4h) treatment. The indicated proteins in co-IP samples or whole cell lysates (WCL) were measured by IB.

(C-G) Co-IP experiments were performed using anti-Myc (C, E) and anti-HA (D, F, G) antibodies in PC9 (F) and H1299 (C, D, E, G) cells with the indicated vectors transfected before erastin (5 µM, 4h) treatment. The TFCP2-Myc (C, E) and YAP-HA (D, F, G) level in each co-IP samples was adjusted to the same protein content. The indicated proteins in co-IP samples or WCL were measured by IB.

(H) Co-IP experiments were performed using anti-YAP in PC9-based GGG cells pretreated with or without Dox (1 μg/ml) and EGCG (5 μM) for 24 h before further treated with erastin (10 μM, 24h). The YAP level in each co-IP samples was adjusted to the same protein content. The indicated proteins in co-IP samples or WCL were measured by IB.

(I) Co-IP experiments were performed using anti-YAP in PC9 cells pretreated with or without EGTA (0.1 mM) for 8 h before further treated with erastin (10 μM, 24h). The YAP level in each co-IP samples was adjusted to the same protein content. The indicated proteins in co-IP samples or WCL were measured by IB.

(J) Co-IP experiments were performed using anti-YAP in *WT* and *ADCY10^-/-^* PC9 cells treated with erastin (10 μM, 24h). The YAP level in each co-IP samples was adjusted to the same protein content. The indicated proteins in co-IP samples or WCL were measured by IB.

(K) Co-IP experiments were performed using anti-YAP in PC9 cells pretreated with or without H89 (10 μM) or Rp-cAMPs (200 μM) for 8 h before further treated with erastin (10 μM, 24h). The YAP level in each co-IP samples was adjusted to the same protein content. The indicated proteins in co-IP samples or WCL were measured by IB.

(L) Co-IP experiments were performed using anti-YAP in PC9 cells with or without ectopically expressed XBP1s before further treated with erastin (10 μM, 24h). The YAP level in each co-IP samples was adjusted to the same protein content. The indicated proteins in co-IP samples or WCL were measured by IB. XBP1s was measured by semi-RT-qPCR via agarose gel electrophoresis.

(M) Co-IP experiments were performed using anti-YAP in PC9 cells pretreated with or without glucose (25 mM), GlcN (5 mM) or GlcNAc (5 mM) for 8 h before further treated with erastin (10 μM, 24h). The YAP level in each co-IP samples was adjusted to the same protein content. The indicated proteins in co-IP samples or WCL were measured by IB.

All the images are representative ones of 3 independent experiments.

**Figure S2.**


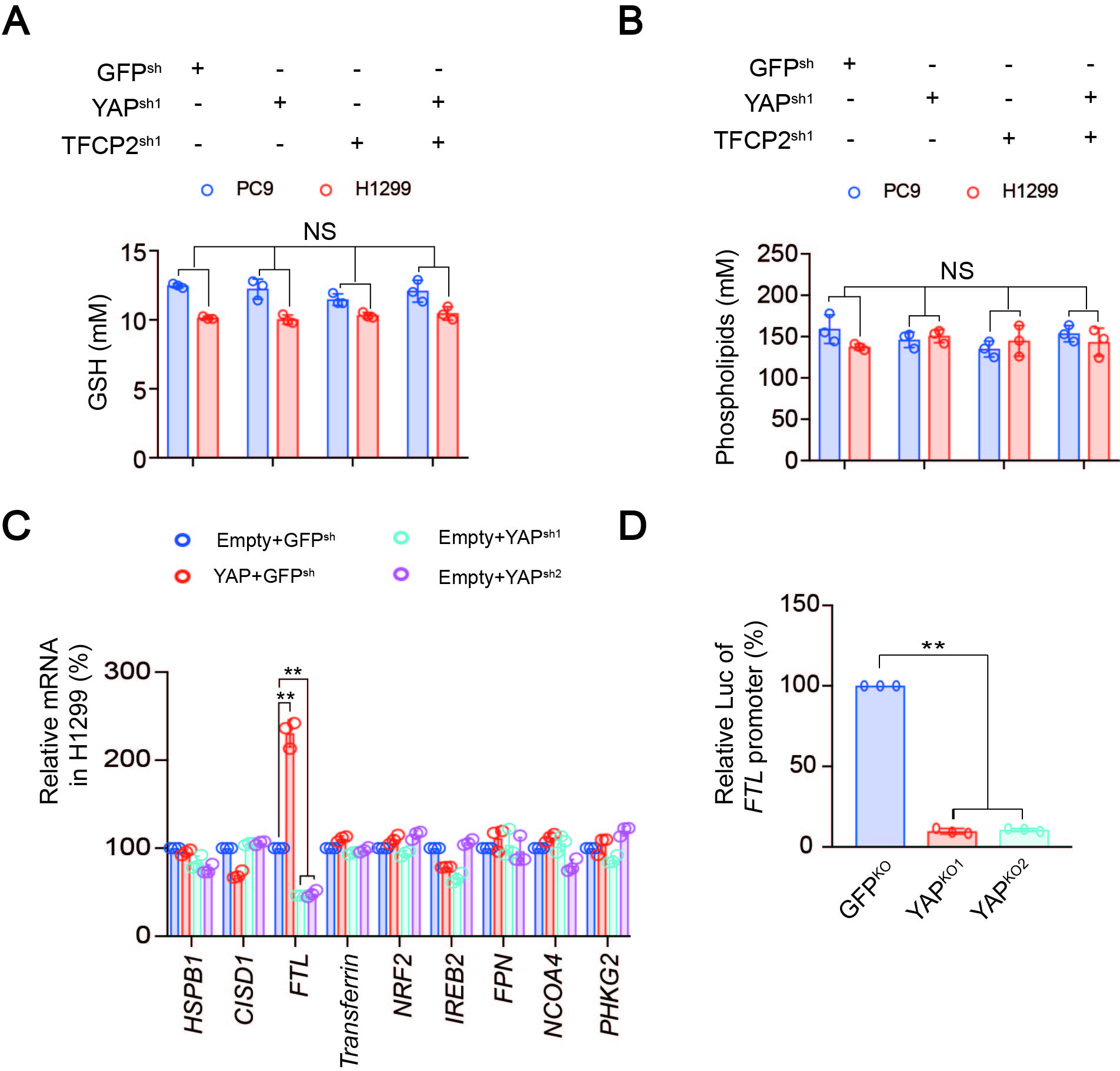


**Figure S2. Supplementary to Figure 2.**

(A-B) GSH and phospholipids were measured in control cells and PC9 or H1299 cells with YAP and TFCP2 individually or simultaneously knocked out.

(C) The indicated mRNA levels measured in control cells and H1299 cells with YAP overexpression or knockdown.

(D) *FTL* promoter luciferase (Luc) activity measured in control cells and PC9 cells with YAP knockout.

The data are shown as the mean ± SD from three biological replicates. **P < 0.01 indicates statistical significance. Data in A-D were analyzed using a one-way ANOVA test.

**Figure S3.**


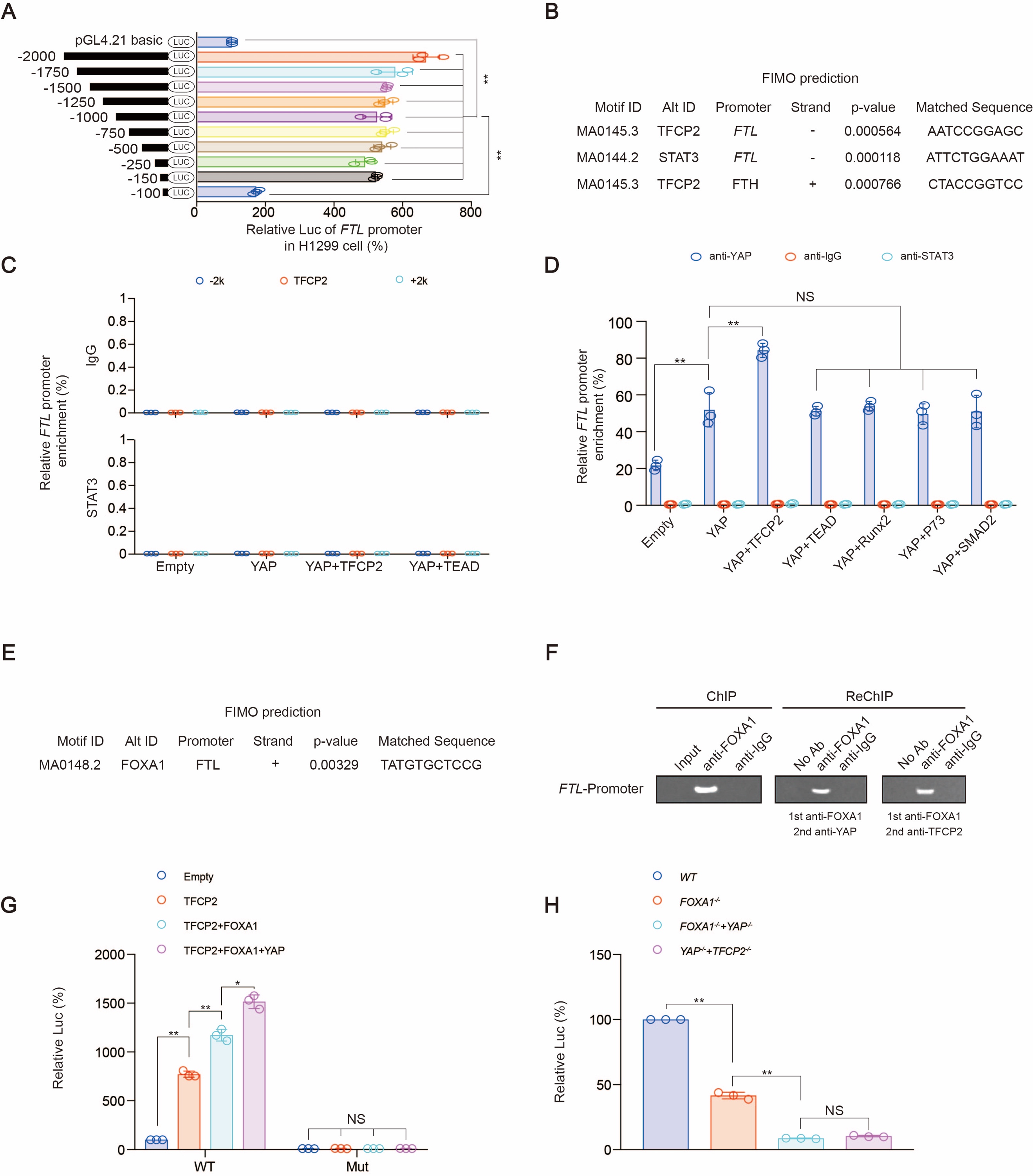


**Figure S3. Supplementary to Figure 3.**

(A) The indicated regions of *FTL* promoter Luc activity measured in H1299 cells.

(B) Localization of TFCP2 or STAT3 motifs in *FTL* promoter was predicted using FIMO tool.

(C) Enrichment of IgG and STAT3 at the indicated region around the *FTL* promoter in control cells and PC9 cells overexpressing YAP with TFCP2 or TEAD.

(D) Enrichment of YAP, STAT3, or non-specific IgG at the indicated region around the *FTL* promoter in control cells and PC9 cells overexpressing YAP with TFCP2, TEAD, Runx2, P73 or SMAD2.

(E) Localization of FOXA1 motifs in *FTL* promoter was predicted using FIMO tool.

(F) ChIP and Re-ChIP assays were used to analyze the co-occupancy of FOXA1, YAP and TFCP2 in PC9 cells by using indicated antibodies.

(G) WT or Mut *FTL* promoter Luc activity measured in control cells and PC9 cells overexpressing TFCP2 with or without FOXA1 and YAP.

(H) WT *FTL* promoter Luc activity measured in *WT* cells and PC9 cells with indicated genes knocked out.

The data are shown as the mean ± SD from three biological replicates. *P<0.05, **P < 0.01 indicates statistical significance. Data in A, D, G, H were analyzed using a one-way ANOVA test.

**Figure S4.**


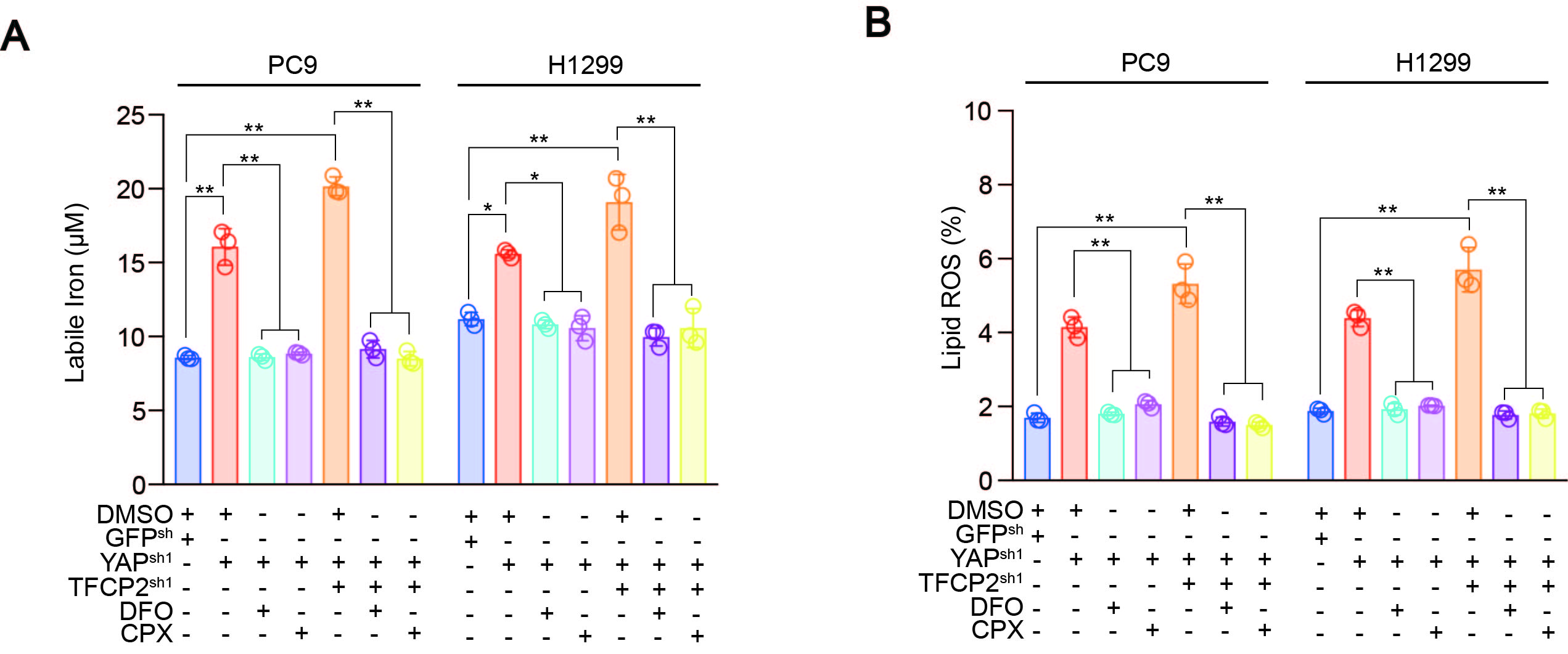


**Figure S4. Supplementary to Figure 4.**

(A, B) Labile iron and lipid ROS generation were measured in PC9 and H1299 cells with or without YAP and TFCP2 knockdown before further treated with DFO (80 μM, 12h) or CPX (10 μM, 12h) as indicated.

The data are shown as the mean ± SD from three biological replicates. **P < 0.01 indicates statistical significance. Data in A, B were analyzed using a one-way ANOVA test.

**Figure S5.**


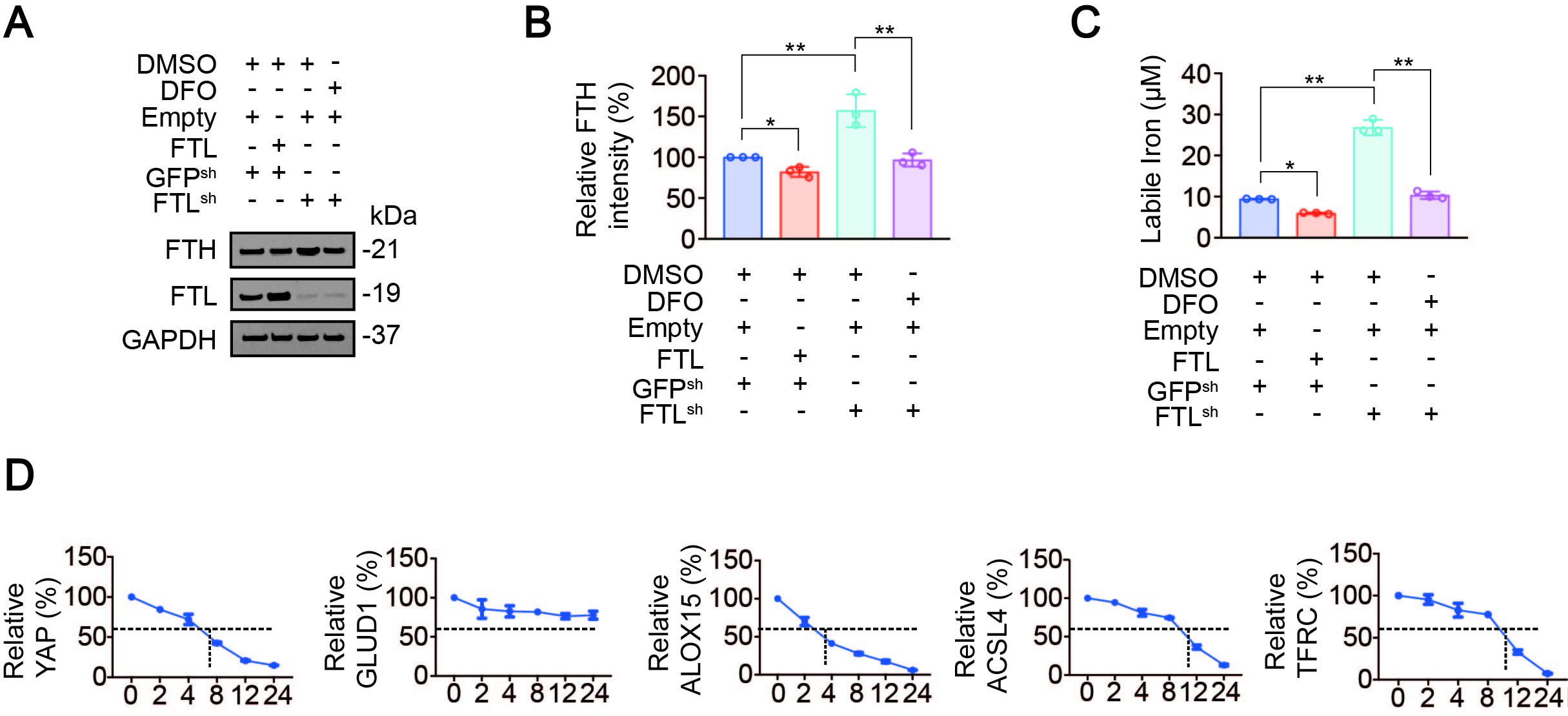


**Figure S5. Supplementary to Figure 5.**

(A-C) FTH and FTL expression (A-B) and labile iron (C) were measured in PC9 cells with or without FTL overexpression or knockdown before further treated with DFO (80 μM, 12h). The level of FTH was normalized to that of GAPDH, and the normalized level of FTH in DMSO-treated PC9 cells was arbitrarily set to 1 (B).

(D) The relative protein levels of indicated proteins as measured in Figure 5J were shown as normalized to that of GAPDH, and the “0 h” points were arbitrarily set to 100%.

The data are shown as the mean ± SD from three biological replicates. *P<0.05, **P < 0.01 indicates statistical significance. Data in B, C were analyzed using a one-way ANOVA test.

**Figure S6.**


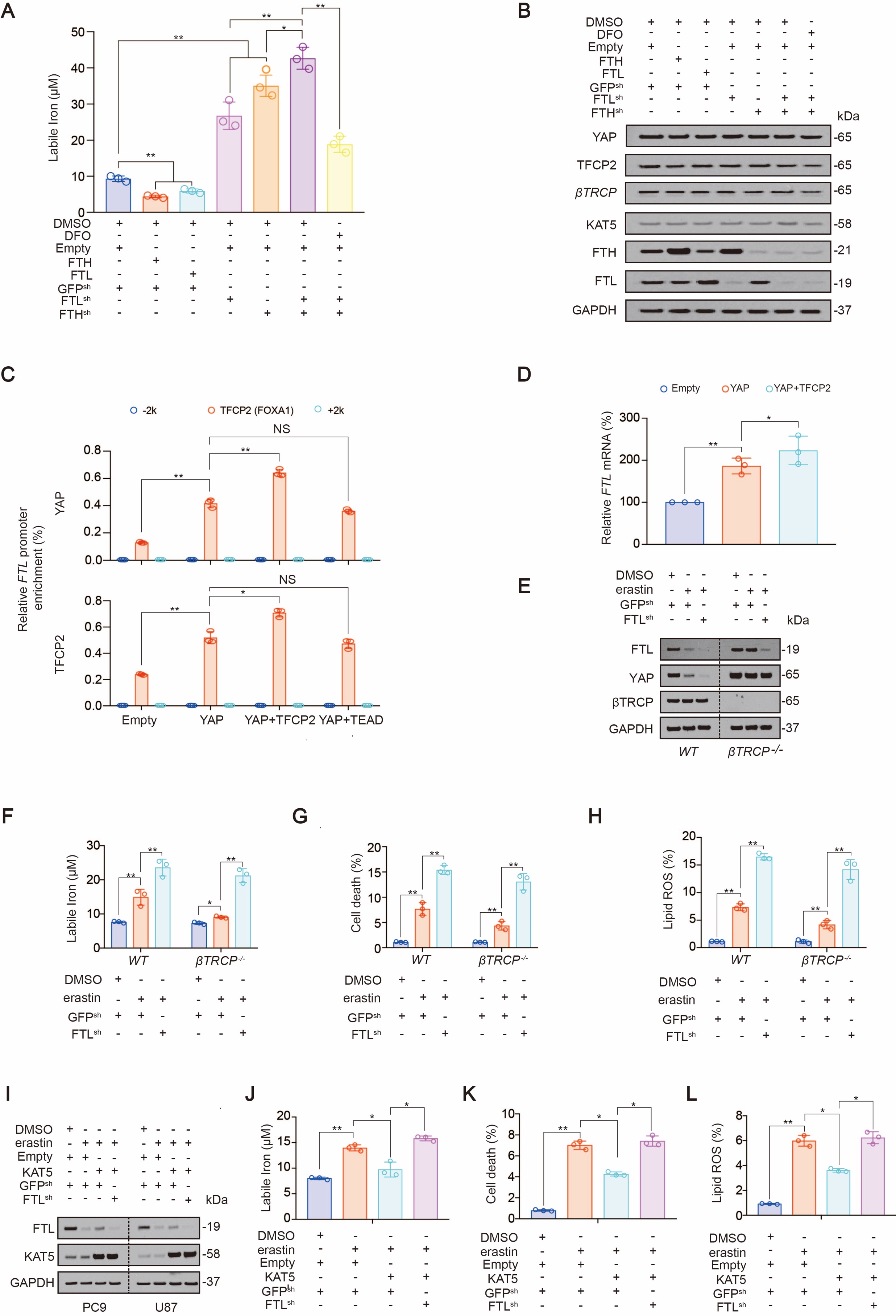


**Figure S6. Supplementary to Figure 6.**

(A-B) Labile iron concentration and indicated proteins were measured in PC9 cells with or without FTL or FTH overexpressed or knocked down before further treated with DFO (80 μM, 12h).

(C) Relative FTL mRNA level was measured in U87 cell overexpressed YAP with or without TFCP2.

(D) Enrichments of YAP and TFCP2 at -2k, TFCP2 (FOXA1)-binding motif or 2k regions within the *FTL* promoter were calculated as the percentage of input chromosomal DNA via ChIP using the corresponding antibodies in U87 cells with overexpressing YAP or TEAD.

(E) FTL, YAP and βTRCP expressions were measured in *WT* or *βTRCP^-/-^* U87 cells with or without FTL knockdown followed by erastin (10 µM, 24h) treatment.

(F-H) Labile iron, cell death and lipid ROS generation were measured in U87 cells with the same treatment as that in panel E. But the treating time was 24 or 16h.

(I) FTL and KAT5 expressions were measured in PC9 or U87 cells with or without KAT5 overexpression or FTL knockdown before further treating with erastin (10 µM, 24h).

(J-L) Labile iron, cell death and lipid ROS generation were measured in U87 cells with the same treatment as that in panel I. But the treating time was 24 or 16h.

The data are shown as the mean ± SD from three biological replicates. *P<0.05, **P < 0.01 indicates statistical significance. Data in A, C, D, F-H, J-L were analyzed using a one-way ANOVA test.

**Figure S7.**


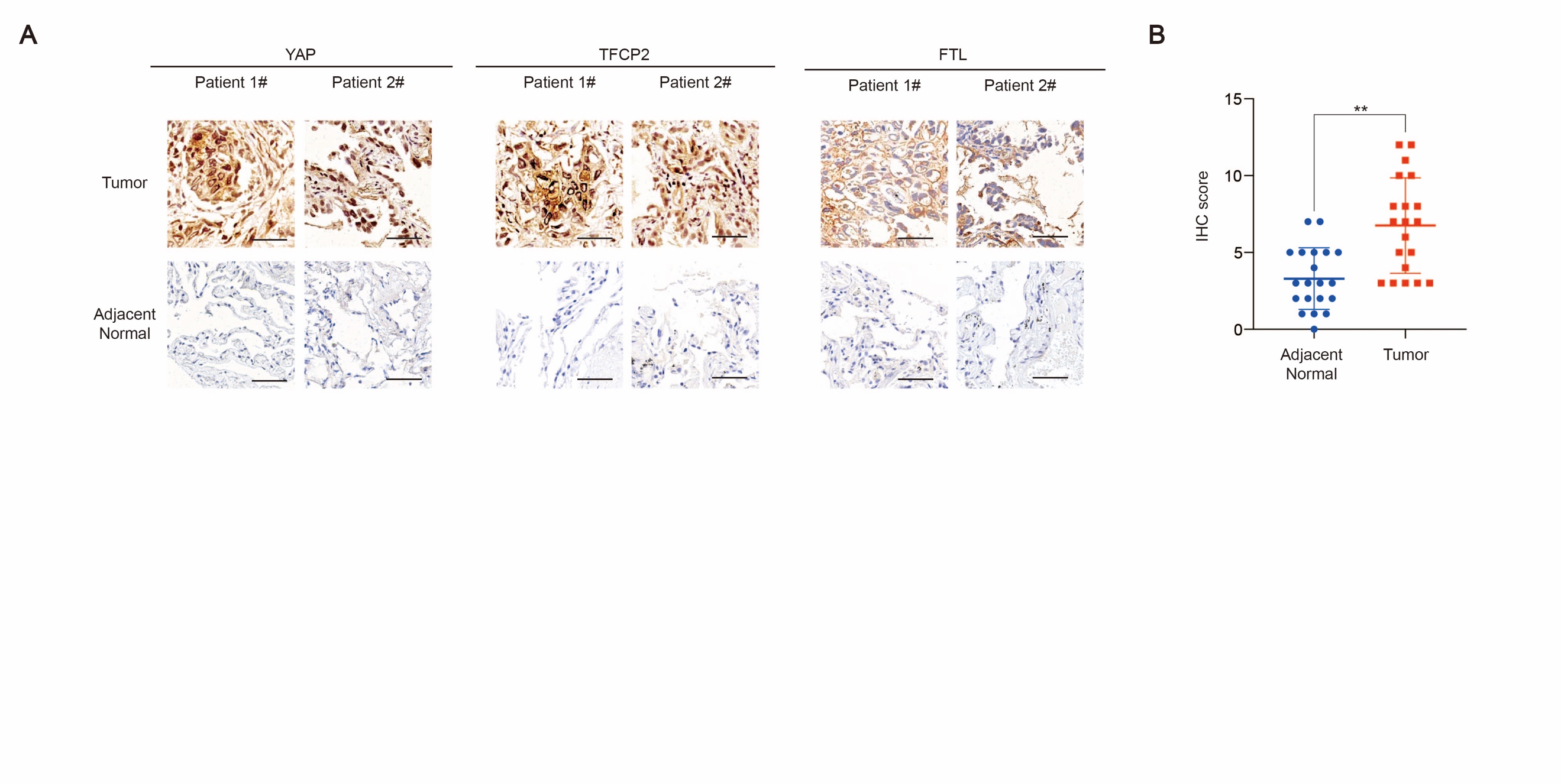


**Figure S7. Supplementary to Figure 7.**

(A-B) Representative IHC images of YAP, TFCP2 and FTL expression in tissues of 2 LUAD patients were shown, scale bar, 100 μm (A). IHC scores for 20 LUAD patient tissues and their adjacent normal tissues were shown, and analyzed by student’s t test (**, P<0.05, B).

**Figure S8.**


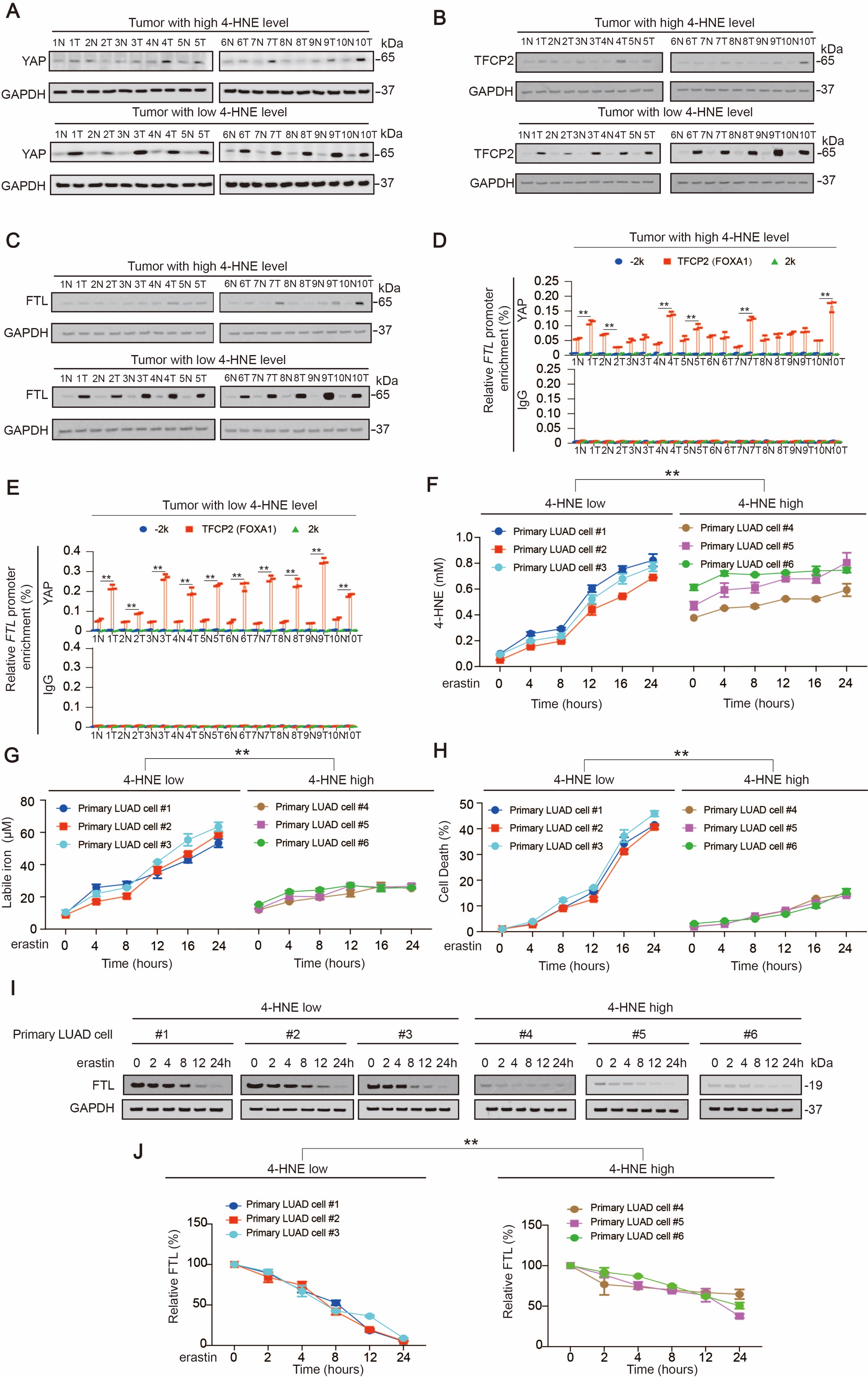


**Figure S8. Supplementary to Figure 7.**

(A-C) YAP (A), TFCP2 (B) and FTL (C) expressions in 10 paired LUAD tumor and adjacent normal tissues with high 4-HNE level (up) or low 4-HNE level (down) was measured using indicated antibodies by IB.

(D-E) Enrichment of YAP or non-specific IgG at the indicated region around *FTL* promoter in 10 paired LUAD tumor and adjacent normal tissues with high 4-HNE level (D) or low 4-HNE level (E).

(F-J) The 4-HNE (F), labile iron (G), cell death (H) and FTL expression (I-J) were measured in Primary LUAD cell #1-#3 (4-HNE low cell) and #4-#6 (4-HNE high cell) after erastin treatment (10 µM) for indicated hours. The relative protein levels of indicated proteins (I) were shown as normalized to that of GAPDH, and the “0 h” points were arbitrarily set to 100% (J).

The data are shown as the mean ± SD from three biological replicates (including IB). **P < 0.01 indicates statistical significance. Data in D, E were analyzed using a student’s t test. Data in F-H and J were analyzed using a two-way ANOVA test.

**Supplementary Table S1. Information of primers, shRNA, sgRNA and probes for EMSA were listed.**

| **Primers used for qPCR** |  |
| --- | --- |
| Name | 5'-3' |
| YAP-qPCR-F | CCTCGTTTTGCCATGAACCAG |
| YAP-qPCR-R | GTTCTTGCTGTTTCAGCCGCAG |
| TFCP2-qPCR-F | CCTTGCATTGCCCATTTTTAAGC |
| TFCP2-qPCR-R | GGAAGTTCTCCAAGTTTCCTATTGTC |
| CISD1-qPCR-F | ATGGAGGATTTGGGAGATAAAG |
| CISD1-qPCR-R | ATCAGGTAACTTCACGACAAG |
| FTL-qPCR-F | GGAGACTCACTTCCTAGATGA |
| FTL-qPCR-R | TAGGCAGAAGCCCTATTACTT |
| NRF2-qPCR-F | CATTCCTGAGTTACAGTGTCT |
| NRF2-qPCR-R | GGACTACAGTTACCTACTTCTT |
| IREB2-qPCR-F | CAGATTCTTGAGTCCATTGTC |
| IREB2-qPCR-R | GCATTACTTAGTAGAGCAACAG |
| PHKG2-qPCR-F | GTGAAGATTATGGAAGTGACAG |
| PHKG2-qPCR-R | GCTAGAAGACTCGTAGGAATC |
| HSPB1-qPCR-F | CAGTCCAACGAGATCACCATC |
| HSPB1-qPCR-R | ATCCGGGCTAAGGCTTTACTT |
| Transferrin-qPCR-F | ACAGAAGCGAGTCCGACTGTGCTC |
| Transferrin-qPCR-R | GGGACCATCGGATGGAATGAC |
| NCOA4-qPCR-F | TGTCTTACTCATCAACTGGAGTGTACCC |
| NCOA4-qPCR-R | ATCAAGTGCTCAGGAATTTGAATGG |
| FPN-qPCR-F | GCAAAATTCCTTCTCTACCTTGGTC |
| FPN-qPCR-R | GGCCACTTTAAGTCTAGCATTCTTG |
| FTH-qPCR-F | AATCTATCCAGGCTATCTTCC |
| FTH-qPCR-R | CCCTTAGTTCTATCTGAATCCA |
| GAPDH-qPCR-F | ATCATCCCTGCCTCTACTGG |
| GAPDH-qPCR-R | GTCAGGTCCACCACTGACAC |
| XBP1s-qPCR-F | AGGAGTTAAGACAGCGCTTGGGG |
| XBP1s-qPCR-R | AATACCTGCACCTGCTGCGGACTCAGCAGA |
| TFRC-qPCR-F | ACCATTGTCATATACCCGGTTCA |
| TFRC-qPCR-R | CAATAGCCCAAGTAGCCAATCAT |
| ACSL4-qPCR-F | ACTGGCCGACCTAAGGGAG |
| ACSL4-qPCR-R | GCCAAAGGCAAGTAGCCAATA |
| ALOX15-qPCR-F | GGGCAAGGAGACAGAACTCAA |
| ALOX15-qPCR-R | CAGCGGTAACAAGGGAACCT |
| GLUD1-qPCR-F  GLUD1-qPCR-R | GGGGGTGCTAAAGCTGGTGTTAAG  GGTATCAGCGATCCAGGACATCTC |
|  |  |
|  |  |
| **Target sequence for shRNA** |  |
| Name | 5'-3' |
| FTL-sh | GCTCCAGAATAACTGCCCTTT |
| FTH-sh | GCCTCGGGCTAATTTCCCATA |
|  |  |
| **Target sequence for sgRNA** |  |
| Name | 5'-3' |
| YAP-KO1 | GGACTCGGAGACCGACCTGG |
| YAP-KO2 | GCAGCAGAATATGATGAACT |
| TFCP2-KO1 | GCCGACGAAGTGATTGAATC |
| TFCP2-KO2 | GTTATTGACATACGTGATCT |
| KAT5-KO | GCGTGAAGGACATCAGTGGC |
| FOXA1-KO | GTAGTAGCTGTTCCAGTCGC |
|  |  |
| **Primers used for promoter analysis** |  |
| Name | 5'-3' |
| WT (-2000) -FTH-promoter-F | ATGCGGTACCAGCTAATGAGGTGTCTCTATGCTGTC |
| WT-FTH-promoter-R | ATGCCTCGAGAGCCGCGTCGGCGTCAGGCCCGCCC |
| Mut-FTH-promoter-F | CCGCTCCCGAGCTCCGCCAGAGCGC |
| Mut-FTH-promoter-R | GGAGCTCGGGAGCGGCCGACTGCCTCTGGGACAGCGGTGG |
| WT (-2000)-FTL-promoter-F | ATGCGGTACCCAGGGTCTCTGTACCCCTCTGTC |
| WT (-2000)-FTL-promoter-R | ATGCCTCGAGGAGGGGACGTGGCTAGGGCGGCTTCTTTTATG |
| Mut-FTL-promoter-F | GGTCAGGCACGGCCTTCGGCCCCGC |
| Mut-FTL-promoter-R | AGGCCGTGCCTGACCGCACATAGGAGTCTCAGCCCCCCGC |
| -1750-FTL-promoter-F | ATGCGGTACCCGGGCCCCACAGTCAACGGGGTGC |
| -1500-FTL-promoter-F | ATGCGGTACCGATTTGGGTCTGAGCCAGGTCTAGTTC |
| -1250-FTL-promoter-F | ATGCGGTACCGGCAGGGCCTGACCTCACCCCAC |
| -1000-FTL-promoter-F | ATGCGGTACCGACACGAGGGCAACTTCAGAGAC |
| -750-FTL-promoter-F | ATGCGGTACCCTACCTCATACAATGCTCCTCCTG |
| -500-FTL-promoter-F | ATGCGGTACCCCAAGGATCCGGAGACTCCTTATCTCG |
| -250-FTL-promoter-F | ATGCGGTACCTGCATTTCCAGAATCAGCCCCAGG |
| -150-FTL-promoter-F | ATGCGGTACCGGGGCTGAGACTCCTATGTGC |
| -100-FTL-promoter-F | ATGCGGTACCCCGCCTCCTGCCACCGCAGATTGG |
|  |  |
| **Probes used for EMSA** |  |
| Name | 5'-3' |
| WT-TF-FTL-F | TATGTGCTCCGGATTGGTCAGG |
| WT-TF-FTL-R | CCTGACCAATCCGGAGCACATA |
| Mut-TF-FTL-F | CTCCTATGTGGGCACGGCCTTCG |
| Mut-TF-FTL-R | CGAAGGCCGTGCCCACATAGGAG |
|  |  |
|  |  |
| **Primers used for ChIP-qPCR** |  |
| Name | 5'-3' |
| FTH-(-2k)-ChIP-F | AGCTAATGAGGTGTCTCTATGCTGTCTC |
| FTH-(-2k)-ChIP-R | GCACTCCAGCCTGGAAGACAAAAC |
| FTH-(TFCP2)-ChIP-F | CTCCTCGCAGCTTCCCTCCAACTCC |
| FTH-(TFCP2)-ChIP-R | TCGCGCGCTCTGGCGGA |
| FTH-(2k)-ChIP-F | CACTGGCTGTAAGTTTTCTGTGCAG |
| FTH-(2k)-ChIP-R | TCAAAGCACAGCATGAAGGAATC |
| FTL-(-2k)-ChIP-F | CAGGGTCTCTGTACCCCTCTGTCTC |
| FTL-(-2k)-ChIP-R | ATACCTCAGCCCAGAGACCTTTAG |
| FTL-(TFCP2/FOXA1)-ChIP-F | ACACGCTGGCGCTACAGGCGCGTGACTT |
| FTL-(TFCP2/FOXA1)-ChIP-R | TAGGGCGGCTTCTTTTATGGTGCGCCG |
| FTL-(2k)-ChIP-F | GCCTGGCTGTTAACTGTCCCTCCCAG |
| FTL-(2k)-ChIP-R | TGACTAGCTGGGATTACAGGTATGAG |
